# A Middleware with Comprehensive Quality of Context Support for the Internet of Things Applications

**DOI:** 10.3390/s17122853

**Published:** 2017-12-08

**Authors:** Berto de Tácio Pereira Gomes, Luiz Carlos Melo Muniz, Francisco José da Silva e Silva, Davi Viana dos Santos, Rafael Fernandes Lopes, Luciano Reis Coutinho, Felipe Oliveira Carvalho, Markus Endler

**Affiliations:** 1 Programa de Pós-Graduação em Engenharia de Eletricidade (PPGEE), Centro de Ciências Exatas e Tecnologia (CCET), Universidade Federal do Maranhão, 65085-580 São Luís, Brazil; lcmuniz@lsdi.ufma.br (L.C.M.M.); fssilva@lsdi.ufma.br (F.J.d.S.e.S.); davi.viana@lsdi.ufma.br (D.V.d.S.); rafaelf@lsdi.ufma.br (R.F.L.); lrc@deinf.ufma.br (L.R.C.); 2 Instituto Federal do Maranhão (IFMA), Av. dos Curiós, S/N, Vila Esperança, 65095-460 São Luís, Brazil; 3 Department of Informatics, Pontifícia Universidade Católica do Rio de Janeiro (PUC-Rio), 22453-900 Rio de Janeiro, Brazil; ocfelipe@inf.puc-rio.br (F.O.C.); endler@inf.puc-rio.br (M.E.)

**Keywords:** context aware applications, Internet of Things, middleware, Quality of Context

## Abstract

Context aware systems are able to adapt their behavior according to the environment in which the user is. They can be integrated into an Internet of Things (IoT) infrastructure, allowing a better perception of the user’s physical environment by collecting context data from sensors embedded in devices known as smart objects. An IoT extension called the Internet of Mobile Things (IoMT) suggests new scenarios in which smart objects and IoT gateways can move autonomously or be moved easily. In a comprehensive view, Quality of Context (QoC) is a term that can express quality requirements of context aware applications. These requirements can be those related to the quality of information provided by the sensors (e.g., accuracy, resolution, age, validity time) or those referring to the quality of the data distribution service (e.g, reliability, delay, delivery time). Some functionalities of context aware applications and/or decision-making processes of these applications and their users depend on the level of quality of context available, which tend to vary over time for various reasons. Reviewing the literature, it is possible to verify that the quality of context support provided by IoT-oriented middleware systems still has limitations in relation to at least four relevant aspects: (i) quality of context provisioning; (ii) quality of context monitoring; (iii) support for heterogeneous device and technology management; (iv) support for reliable data delivery in mobility scenarios. This paper presents two main contributions: (i) a state-of-the-art survey specifically aimed at analyzing the middleware with quality of context support and; (ii) a new middleware with comprehensive quality of context support for Internet of Things Applications. The proposed middleware was evaluated and the results are presented and discussed in this article, which also shows a case study involving the development of a mobile remote patient monitoring application that was developed using the proposed middleware. This case study highlights how middleware components were used to meet the quality of context requirements of the application. In addition, the proposed middleware was compared to other solutions in the literature.

## 1. Introduction

Context data is any information that can be used to characterize the situation of an observed entity (e.g., person, device, system) [1]. Context-aware applications use such information to provide relevant services to their users, with minimal human intervention [2]. The requirements of a context-aware application varies according to its domain and goals, and the discovery of context data sources and services is an essential mechanism that allows applications to be informed of the available context information.

The Internet of Things (IoT) is a research field that integrates context awareness, mobile computing, wireless sensor networks, communication protocols and devices with embedded sensors and/or actuators. This integration aims to generate a global dynamic system that collects and distributes context information provided by thousands of smart objects for diverse applications [3]. IoT applications are being developed in several areas such as health, transportation, commerce, industry, and agriculture.

In many cases, due to memory and processing restrictions, smart objects do not have connectivity to medium and long range networks. They do not implement a Transfer Control Protocol/Internet Protocol (TCP/IP) protocol stack, making them unable to access the Internet with their own resources. However, these smart objects are able to send their context data to a local gateway, using some kind of short-range communication technology, such as Bluetooth or ZigBee. Upon receiving this data, the gateway will be in charge of forwarding it to the Internet through network technologies such as Wifi or 3G/4G. An IoT extension, called Internet of Mobile Things (IoMT) [4], conceives situations where smart objects and IoT gateways can move or be moved with great flexibility. Wearable or portable devices, robots, and vehicles are some examples of moving objects. In this scenario, conventional tablets and smartphones can act as mobile system gateways, promoting discovery and opportunistic connection with smart objects all around.

One of the factors that have a significant impact on the behavior of IoT applications and on the quality of experience of their users is the Quality of Context (QoC) [5]. In a traditional and very restricted view, QoC expresses only the quality of the information (QoI), excluding from this concept the quality of the distribution service (QoS) and the quality of the devices (QoD) that provide the information [6]. Contrary to this view, some researchs argue that the concept of QoC needs to be updated to encompass both the quality of the information and the quality of the distribution [7], in order to better meet the requirements of context consumers. A good example of a more comprehensive definition of QoC, which has been well accepted in the literature, was proposed by [8]. For these authors, the concept of QoC “indicates the degree of conformity of the context collected by sensors to the prevailing situation in the environment and the requirements of a particular context consumer”. This modern concept of QoC meets the objectives of this work.

The rationale behind this more comprehensive view is that in several cases the quality of the information is affected by the quality of the data distribution service and vice versa. For example, the age that the context information (QoI parameter) will have when it reaches the consumer will depend on the communication delay (QoS parameter) between the producer and the consumer. In another example, the validity time of the context information (QoI parameter) can be used to implement lifespan mechanisms (QoS policy). These mechanism remove from the history/cache those data whose validity time has already expired.

An aspect of QoC is its dynamic variability (it oscillates over time). In the best scenario, the QoC of a selected service provider will improve, staying above the application expectations. In the worst case, the QoC will fall in such a way that, at some point, it will no longer meet the requirements demanded by the application. Several factors can degrade QoC at run time, such as hardware and software failures of sensors, gateways, servers, and communication networks [9,10].

There are several reasons why applications may want to be aware of the available QoC level. For example, QoC can be used as a criteria for consulting and selecting service providers. Thus, applications can search and choose service providers that best suit their context and QoC requirements. QoC can also be a clause to be defined in Service Level Agreements (SLAs), where producers declare context services and the QoC level they can provide, while consumers specify the information of their interest and the required quality level [6].

A possible approach for the development of IoT systems is the one where applications directly connect to the sensors, without any intermediate layer between them and the operating system. However, when a large number of smart objects, technologies, and heterogeneous communication protocols are applied, this approach becomes infeasible. In order to deal with this complexity, several solutions based on a middleware approach have been proposed. Each solution focuses on different aspects of IoT, such as device management, interoperability, portability, context modeling, data distribution, dynamic adaptation, security and privacy, among others. Although some initiatives attempt to address multiple aspects, an ideal IoT middleware solution, able to handle all these challenges, has not yet emerged.

In recent years, some middleware proposals have appeared in order to support the development of context-aware applications with QoC requirements. However, the QoC support provided is not yet fully integrated with existing solutions and also has several gaps [11]. Although research on the development of QoC-supported context middleware is relevant to the construction of context-aware systems, the initiatives are still considered insufficient [12], motivating the continuity of efforts.

This article presents two main contributions. The first contribution consists of a literature review regarding context middleware aimed at IoT applications with integrated QoC support. It is important to emphasize that, although there are works whose objective is the survey of middleware systems of context management, a comparative analysis focused on the QoC support offered by the different tools, considering different aspects, is still lacking. The second is the presentation and evaluation of a new middleware for the development of applications for IoT with comprehensive support for interaction with physical sensors, reliable distribution of context data, QoC provisioning and monitoring. In addition to being submitted to quantitative evaluation, the middleware was validated through a case study involving the development of a mobile application with QoC requirements aimed at monitoring patients using wearable sensors. In order to reinforce the contributions of the proposed solution to the state of the art, a comparative analysis is promoted between this new middleware and those found in the literature consulted.

The remainder of this paper is divided as follows. Section 2 presents the state of the art related to this work, giving greater emphasis to the analysis of the main proposals of context middleware with QoC support known in the literature. Section 3 shows an overview of the proposed middleware, its requirements, architecture and components. A case study in which the middleware can contribute significantly in the development of a solution is also shown. The functionalities and implementation aspects of the solution presented in case study are illustrated in Section 4. Section 5 describes the middleware performance evaluation experiments and discuss their results. The Section 6 presents a discussion about the advances obtained with proposed solution in relation to the other middleware systems with QoC support presented in Section 2. Finally, Section 7 presents conclusions and future work.

## 2. State of the Art of Middleware with QoC Support

The state of the art related to Context-Aware systems and Internet of Things and encompasses works with different objectives.

Cavalcante et al. [13], Breivold [14], Weyrich and Ebert [15] analyzed different reference architectures for IoT. A reference architecture defines an initial abstract set of building blocks for IoT environments, taking into account all the requirements of these environments [13]. A reference architecture is the basis for building concrete architectures. Some of the main reference architectures analyzed in these paper are: Reference Architecture Model for Industrie 4.0 (RAMI4.0) [16], Industrial Internet Reference Architecture (IIRA) [17], IoT Architectural Reference Model (IoT-ARM) [16], Arrowhead Framework [18], WSO2 IoT Reference Architecture [19] and Intel IoT Platform Reference Architecture [20]. Bellasvista et al. [7], Delicato et al. [21], Perera et al. [22], Bandyopadhyay et al. [23] Li et al. [24] and Yürür et al. [25], Blair et al. [26] produced surveys of context-aware systems and IoT. Addressing issues related to Quality of Context (QoC), Buchholz et al. [6], Krause et al. [27] and Manzoor et al. [8] provided motivation and definitions of several QoC parameters. Lei et al. [28], Henricksen et al. [9], Hönle et al. [29] presented the advantages of describing characteristics of the context information using QoC metadata. Gray et al. [30], Buchholz et al. [6], Henricksen et al. [9], Hönle et al. [29], Schmidt [31]. Sheikh [32] and Manzoor [8] focused on the proposition and/or evaluation of QoC metrics. Bu et al. [33], Schmidt [31], Herbscher et al. [34], Chantzara et al. [35], Pawar et al. [36], Neisse et al. [37], Sheikh et al. [32] and Manzoor et al. [38] presented different motivations and situations for the use of QoC in context-aware systems.

However, none of these works focus on providing a deep analysis of context middleware systems with support for QoC provisioning and monitoring. This Section describes the middleware systems that, to the best of our knowledge, can be considered the most relevant ones in respect to QoC support: AWARENESS [39], COntext entitieS coMpositiOn and Sharing(COSMOS [40,41], COntext Provisioning for ALl (COPAL) [42,43], INCOME [11,44,45] and Scalable context-Aware middleware for mobiLe EnviromentS (SALES) [46,47,48,49]. After the middleware descriptions, an analysis of their limitations and open research issues are presented.

### 2.1. AWARENESS

AWARENESS [39] is an infrastructure for the development of adaptive context-aware mobile applications. The middleware architecture is divided into two layers: application and infrastructure. The application adaptation mechanism is based on Event-Condition-Action rules [50] that are executed in the infrastructure layer, which incorporates an ECA Engine. When an application defined event of interest is detected, the software infrastructure triggers the ECA related action that will be performed by the application itself. A peculiar feature of this middleware is that applications can interact with the infrastructure and retrieve context data in two distinct ways: synchronous (request/response) and asynchronous (publication/subscription). AWARENESS also distinguishes two forms of service discovery: instantaneous (or active) and subscribed (or passive). In the first, the client issues a request and a discovery service immediately returns a response containing the available services satisfying the specified criteria. In the second, the discovery service will notify the client about available services meeting the specified criteria as they are discovered withing a client specified time period. In relation to QoC provisioning, context sources can provide the following metadata that characterizes QoI: *accuracy*, *probability of correctness*, *trustworthiness*, and *up-to-dateness*. The user can specify, through privacy policies, the type of context information, the recipient, and the QoC level he/she wants to share. In some cases, the defined privacy policy requires the direct consent of the user, which releases the information on a case-by-case basis. With regard to quality of service, the middleware provides *persistence policies* and *data history*. In the paper describing AWARENESS a case study involving a remote monitoring application of patients with epilepsy is presented. However, the work does not present the QoC requirements of this application.

### 2.2. COSMOS

COSMOS [40] is a context management framework for ubiquitous environments. All the system entities are modeled and implemented from a basic structure, called *context node*, organized hierarchically. Communication within the hierarchy can be bottom-up (notification) or top-down (observation). The COSMOS architecture is conceptually divided into three layers: lower, intermediate and higher. In the lower layer reside context collectors, which provide raw data for the upper layers. At the middle layer there are context processors, which filter and aggregate the collected data, even though the system does not provides the implementation of data processing techniques. In the upper layer reside the context nodes responsible for the situation inference, which may trigger some kind of application adaptation. COSMOS supports the following quality information parameters: *Up-ToDateness*, *Trustworthiness*, *Accuracy*, *Precision*, *Security* and *Completeness*. However, according to the authors, new parameters can be added. The disclosure of the QoC can be done in two ways: (1) together with the information; or (2) separately from the information. In the first mode, all information is enriched with QoC. This mode is useful for applications interested in both the information itself and the QoC associated with it. In addition, it allows the filtering of information based on QoC but imposes a higher computational cost. In the second mode, the QoC is sent in separate messages, periodically or through an application request. In this case, the QoC information that is sent may correspond to the last computed QoC or to an average. This approach consumes fewer resources and is compatible with applications that use COSMOS versions without QoC support. The extended version of COSMOS with QoC support was evaluated through a case study involving an e-commerce application. The QoC requirements of the application served by the middleware are: Trustworthiness, Up-to-Dateness and Precision.

### 2.3. COPAL

COPAL [42,43] (http://www.infosys.tuwien.ac.at/m2projects/sm4all/copal/downloads.html) is a context management middleware for adaptive applications. The middleware provides broad support for event specification and processing (e.g., filtering, aggregation, summarization, etc.) based on the use of Complex Event Processing (CEP) [51] rules. The COPAL architecture is divided into three layers: device services, COPAL core, and context-aware services. The device services layer describes the devices and sensors with which the middleware interacts. The device discovery and connection are based on the UPnP protocol [52]. From COPAL’s point of view, each device is a publisher. The COPAL core layer concentrates the responsibilities related to the registration of components and services, processing of context queries, event processing and execution of actions. The Context-aware Services layer contains listeners that applications use to be notified when their context queries are met. The context data exchange between publishers and context-aware applications is supported by an event processing service implemented with the Esper CEP Engine (http://esper.codehaus.org). In terms of quality of information, COPAL supports the following parameters: *Source Location*, *Authorization*, *Freshness*, *Trustworthiness*, *Precision* and *Time-to-Live*. Regarding quality of the distribution service, COPAL supports policies that manage the priority of event delivery, as well as mechanisms that discard those events (stored or not in a history) as soon as the data lifetime expires. One of the main limitations of COPAL is the fact that the distribution of context data is only local. However, the authors plan for the implementation of a distributed architecture. Programmers using the COPAL middleware have the flexibility to develop applications using both an Application Programming Interface (API)-based model and a Domain Specific Language (DSL)-based model. The only type of evaluation presented in the articles describing the middleware is restricted to comparing the size of the files and the number of lines of code obtained when using both programming models in the implementation of a smart home monitoring application.

### 2.4. INCOME

INCOME [11,44,45] is a distributed context middleware with content-based data routing. The processing and consumption of context data can be distributed on servers or mobile devices with limited resources. The processing functions (e.g., filtering, aggregation, inference) are defined using JavaScript. INCOME support for application development provides four main components: Context Collector, Context Capsule, Context-Aware Application, and Brokers. Context Collectors are responsible for encapsulating the complexity of raw data acquisition from physical sensors. Context Capsules are responsible for processing the collected data and transforming it into more high-level context information. This information can be made available to other Context Capsules and/or consumer applications. Therefore, this component can act as a context producer and/or consumer. The Context-Aware Application component, as the name suggests, is used by the consumer application to query and receive context data. Brokers are components that act as intermediaries between producers and consumers. These brokers are organized in a network overlap, in which each customer (producer or consumer) can connect to a single broker at a time. The distribution mechanism was implemented using a framework called MUltiscale Distributed Event-Based System (MuDEBS) [53]. The quality of information support provided by INCOME is based on an API that allows producers and consumers to specify their context and QoC offerings and requirements. The middleware supports the following parameters: *Freshness*, *Precision*, *Completeness*, *Accuracy* and *Spatial Resolution*. A peculiar feature of INCOME is that quality of information parameters are implemented using a model-driven approach (The model-driven engineering allows to generate a complete or partial system implementation from the system model [54]), using a framework called Quality of Context Information Model (QoCIM) [55]. INCOME was evaluated through a case study involving an urban pollution monitoring application . The QoC parameters required by the application are: Refresh Rate, Precision and Spatial Resolution.

### 2.5. SALES

SALES [46,47,48,49] defines an infrastructure for the distribution of context data based on QoC. Nodes running a middleware instance are logically organized hierarchically and can communicate with each other to send and/or receive context data a via fixed infrastructure network or an ad hoc mobile network. SALES provides a mobility management mechanism for neighboring nodes. This mechanism promotes the opportunistic discovery of the devices as well as creates groups of nodes that are coordinated by a dynamically elected leader node. Information retrieval is made possible by context queries dissemination. These queries express the context and QoC requirements of the requesting node and can be disseminated both horizontally and vertically in the hierarchy. Upon receiving a query, each node checks the context data stored in its cache and, if there is a positive match, creates a context response containing the requested information. The response is then routed back to the requesting node using a hop-by-hop mechanism. The middleware provides three parameters that determine the quality of the context data distribution: *Freshness*; *Data Retrieval Time* and *Priority*. These parameters are defined by means of a Context Data Distribution Level Agreement (CDDLA). In SALES, there are three user classes whose QoC parameter values are statically defined: (i) Gold: has priority 0 and requires receiving the most recent version of the data in a recovery time of up to 2 s; (ii) Silver: has priority 1 and accepts to receive valid data (even if not the most recent) in a recovery time of up to 4 s; (iii) Bronze: has priority 2 and accepts to receive data already expired in a recovery time of up to 6 s. For data retrieval to occur according to CDDLA, SALES automatically maps QoC parameters to context query parameters. Query parameters specify how the infrastructure will route and treat queries. The SALES distribution service performance was evaluated through experiments. The objectives of the experiments were to measure the retrieval time of the context information the percentage of satisfied context requests in different scenarios.

### 2.6. Analysis and Open Issues

Analyzing the middleware systems with QoC support described, it is possible to see that they cover one or more issues relevant to the development of IoT applications, such as context modeling, data distribution and requirements specification of QoC. However, it is also possible to notice that there are at least four open issues, such as:

- QoC provisioning: the middleware’s ability to provide a significant variety of QoI and QoS parameters, in order to satisfy IoT applications that have multiple QoC requirements;
- QoC monitoring: the middleware’s ability to provide mechanisms that detect variation in context quality at runtime in order to allow context aware applications to dynamically adapt to such oscillations. In this case, the middeware should allow the application itself to define the monitoring events it requires to be notified in real time;
- Heterogeneous physical sensors management: the middleware’s ability to discover device services and to interact with heterogeneous sensors and technologies in a dynamic and opportunistic way. In addition, the middleware must abstract the hardware heterogeneity so that the application does not know the details of the sensor driver implementation;
- Reliable data delivery in mobile environments: the middleware’s ability to ensure delivery of context data, even in situations where applications run in mobile environments with weak or intermittent connectivity or when IP address exchanges happen.

Table 1 summarizes a comparison between AWARENESS, COSMOS, COPAL, INCOME and SALES emphasizing the described open research issues.

In respect to QoC provisioning, INCOME and COSMOS are focused only on the quality of information. AWARENESS and COPAL, while more focused on QoI, also provide some quality service policies. However, QoS support in AWARENESS is limited to persistence and data history. In COPAL, the quality of the distribution is somewhat less limited than the one provided by AWARENESS, since it includes policies for prioritizing event delivery and eliminating data and events that exceed defined lifetimes. SALES, on the other hand, focuses only on the quality of the distribution, supporting three parameters (Time To Live, Data Retrieval Time and Priority). Therefore, the major limitation of these middleware systems in this aspect is that they natively offer few QoI and QoS policies/parameters, and are unsuitable for applications that have multiple QoC requirements.

Regarding QoC monitoring, none of the middleware systems presented provides evidence for the existence of mechanisms that allow context-aware applications to specify events of changes in quality of context so that they are notified of the occurrence of such events in real time. Therefore, one limitation of these middleware systems is that they are not suitable for applications that need to dynamically adapt to QoC variations. Although there are works whose objective is to implement and evaluate QoC monitoring mechanisms [56], since they are not integrated with a context middleware they are not subject of this paper.

Concerning support for management of heterogeneous devices and technologies, most middleware systems presented do not offer any type of service that dynamically manages the discovery, connection, and acquisition of raw data from physical sensors. Almost all solutions are limited to providing only a set of interfaces or abstract classes that facilitate the implementation of sensors adapters, also called wrappers, specific to each device. Wrappers encapsulate the sensor drivers provided by the manufacturer and expose to the upper layers of the middleware, in a standardized and convenient way, all or only some functionalities/services natively implemented by the by the sensor drivers. In other words, the wrapper function is to abstract the hardware heterogeneity. Therefore, IoT application developers often have the task of extending the context middleware and implementing these wrappers, as well as the responsibility of implementing the sensor discovery and interaction mechanisms. COPAL escapes the rule because, besides standardizing the implementation of wrappers, it offers a device management service that is responsible for the sensor discovery and monitoring of device status. The device services description is based on the Universal Plug and Play (UPnP) standard, which can be extended. It is important to note that the device management service offered by the SALES middleware does not apply to sensors, but only to the discovery and acquisition of data from mobile nodes that execute an instance of the middleware.

The support for the reliable delivery of context data in mobile environments is also neglected by the middleware systems presented. AWARENESS and COPAL solutions, for example, do not define a standard network communication technology in the middleware layer, transferring that responsibility to the application programmer. It is therefore natural that these middleware systems fail to address the inherent challenges of communicating in mobile environments. COSMOS, INCOME, and SALES, although they indicate the existence of mechanisms to support distributed communication, also do not explore the implementation of mechanisms that guarantee the reliability of data delivery in unstable networks.

Taking into account the evaluation methodology of each presented middleware system, it is noted that most evaluations are restricted to concept tests based on case studies, without the performance of the middleware being measured. The exception to the rule is the SALES middleware, which presents a performance evaluation of the distribution service. Given the above, it is considered that the presented middleware systems still need to move towards more comprehensive QoC support. It is necessary to extend QoI and QoS provisioning in order to meet the different quality requirements of a wide variety of IoT applications, and it is necessary to extend the middleware systems so that they are able to detect variations of QoC in real time. It is noticeable the need to advance in the provision of sensor management mechanisms capable of dealing with aspects of mobility and volatility of intelligent objects. Similarly, the presented middleware systems still lack mechanisms that guarantee delivery reliability in mobile environments, because for many applications data loss has negative impacts on the quality of service. In addition, most middleware systems still require more consistent performance evaluations.

## 3. Proposed Solution: M-Hub/CDDL

### 3.1. Middleware Overview

Mobile Hub (M-Hub)/Context Data Distribution Layer (CDDL) is a new middleware for context data management (acquisition, processing, and distribution) with broad support for the development of context-aware IoT/IoMT applications that have QoC (QoI and QoS) requirements. This middleware was developed by the Laboratory of Intelligent Distributed Systems (LSDi) of the Federal University of Maranhão (UFMA), in partnership with the Laboratory for Advanced Collaboration (LAC) of the Pontifical Catholic University of Rio de Janeiro (PUC-Rio). The proposed solution combines a mobile gateway (the Mobile Hub [4,57]) for the acquisition of raw data from heterogeneous physical sensors with a CDDL. This layer is also responsible for registering and discovering the available context services, as well as for provisioning and monitoring context information and for ensuring the context data distribution service quality.

In previous work [4,57,58,59,60,61,62,63], M-Hub has used the Scalable Data Distribution Layer (SDDL) as the default mechanism for the dissemination and processing of context data. SDDL uses two distinct communication protocols in the path between context producers and consumers: Mobile Reliable UDP (MR-UDP) and Data Distribution Service (DDS) [64]. MR-UDP is used for communication between mobile clients and servers that act as entry points to the SDDL cloud (SDDL Gateways). MR-UDP implements typical TCP functionality, such as packet delivery and packet retransmission, but on top of a UDP protocol stack [61]. In turn, the DDS is used for communication between the different types of servers running in the SDDL cloud, that is, the inter-node stationary communication inside the cloud. DDS is a fully decentralized and scalable publish/subscribe communication protocol, with support for various QoS policies, such as reliability, deadline, latency, history, ordering, etc. However, the QoS offered by DDS in the cloud does not extend to mobile clients as they use MR-UDP. There is no QoS compatibility between MR-UDP and DDS. Therefore, the use of two distinct protocols is one of the factors that makes SDDL not to offer end-to-end QoS.

Unlike SDDL, CDDL adopts Message Queuing Telemetry Transport (MQTT) [65,66] as the only communication protocol, both local and remote. This decision ensures that even QoS policies based on MQTT, such as reliability, for example, are configured and applied end-to-end. Another advantage of adopting a single protocol is that this eliminates the need to convert messages from one protocol to another. MQTT is a protocol implemented over TCP, which offers different levels of reliability and uses brokers to exchange messages between publishers and subscribers. This protocol presents low communication overhead, making it a good choice for telemetry applications that run on networks with low bandwidth and high latency. In addition, because it is lightweight, MQTT consumes few computational resources and is adequate for use on mobile devices with limited resources [67].

Once connected, MQTT clients can send and receive messages to/from brokers using topics. Upon receiving a message, a broker analyzes the publication topic and forwards the message to one or more online subscribers who have signed the same topic (topic-based filtering). Therefore, a broker prevents MQTT clients from having to establish a direct connection between them so that they can share messages. A priori, brokers do not store any messages received after delivery confirmation to subscribers (which is only necessary in cases where delivery reliability is required), unless the message was marked by the publisher as “retained”. Retained messages will be forwarded to subscribers who were offline at the time these messages were published. Unlike technologies such as JMS, brokers do not maintain message queues for each subscriber.

DDS and MQTT share some common principles, such as parsimony and efficiency, temporal decoupling, and anonymity. However, each protocol has unique characteristics that make it more applicable to certain use cases [67]. In addition to the features already presented, CDDL adopts MQTT because it is an optimized protocol for device-to-cloud communication. SDDL, in turn, adopts DDS for inter-node messaging in the cloud, as it is an optimized protocol for device-to-device communication, which is based on the use of IP multicast for the implementation of global data buses. However, since IP multicast is not available from commercial Internet providers, the potential of DDS, especially QoS policies, can not be fully exploited outside of local networks. While there is the possibility of encapsulating DDS messages in Hypertext Transfer Protocol (HTTP) requests, this type of solution is generally provided by a small group of private companies, which aim to provide communication between DDS applications spread across multiple web domains or that seek interoperability between local DDS applications and remote applications that use other protocols, including MQTT itself. However, even with this possibility, CDDL rules out the use of DDS on mobile clients because that would make the middleware dependent on proprietary solutions whose acquisition involves costs. In favor of MQTT, the availability of open-source MQTT libraries and brokers makes CDDL independent of proprietary solutions.

The proposed solution suggests that developers use M-Hub and CDDL in an integrated way. However, these layers have been designed in such a way that they can be executed independently of one another. This means that the M-Hub can disseminate data using other distribution services (besides the CDDL), and that the CDDL can publish context data collected using other data acquisition services. M-Hub runs on personal mobile devices (e.g., smartphones and tablets) running the Android platform. There are three types of CDDL clients: mobile (for Android devices), desktops (for personal computers, workstations, and servers with a Java Virtual Machine (JVM) installed) and web. Mobile and desktop clients follows the Internet of Things model and use the standard version of the MQTT protocol (3.1.1 specification) [66]. Web clients, in turn, follows the Web of Things (Wot) [68] model and use MQTT as a protocol with WebSockets.

M-Hub/CDDL simplifies the process of developing applications for IoT/IoMT because it abstracts the complexity that is related to mechanisms that are responsible for several phases of the context management process, providing the programmer with functionalities ranging from data acquisition to its delivery to the consumer. For example, sensors discovery and interaction via Wireless Personal Area Network (WPAN) is a complex task, but developers do not have to worry about it as the middleware already supports some WPANs and can be easily extended to support others. Depending on the sensor type, some QoC parameters can be automatically measured without the programmer having to intervene in this process, which is also an advantage. Another task that the middleware simplifies is the distribution of context data. Regardless of the origin of the data, whether local or remote, the programmer can use the same interfaces/methods/abstractions, without having to adopt different programming models. Instead of requiring the programmer to implement mechanisms responsible for managing the quality of the distribution service, M-Hub/CDDL already offers a very broad set of QoS policies. Thanks to them, the programmer simply configures the parameters that determine how, when and where the data will be delivered. Nevertheless, the middleware simplifies operations of context service discovery, filtering, and data monitoring, taking into account QoC, through the use of a very expressive language.

**Figure 1:**
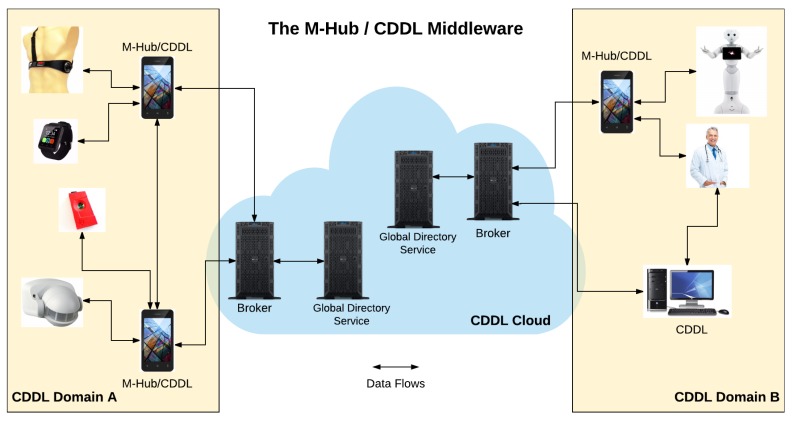
M-Hub/CDDL Infrastructure Overview.

Figure 1 illustrates the use of the M-Hub/CDDL infrastructure in a scenario known as Ambient Assisted Living (AAL). In this scenario, monitored patients use a body sensor network that provides data related to their health status and a smartphone running the M-Hub/CDDL for collecting sensor data and distributing it through brokers located in a cloud. The health sensor data can be delivered to physicians, caregivers and the patient’s family members. This scenario is an example of IoMT, since it assumes unrestricted mobility of wearable/portable sensors, local gateways, and consumer applications running on mobile devices.

AAL applications may have different context requirements. For example, an AAL emergency care application needs to receive monitored patient location data with sufficient accuracy to direct the ambulance service to the exact location he/she is in. Therefore, monitoring the data accuracy or filtering data based on this QoI can be very useful in this case. In AAL systems, event delivery reliability that characterizes an emergency is a fundamental QoS requirement, because if the event notification is not delivered to the monitoring center, no decision and action will be taken, for lack of knowledge of the situation in which the patient is. Therefore, patient monitoring systems need a mechanism that detects the loss of messages and retransmits the information in case of failure to deliver it.

The complexity in the development of context aware applications, such as the AAL, imposes several requirements for the middleware solutions. In order to elucidate the necessary requirements for a better QoC management of by the middleware, a case study of a context-aware application will be presented in the following section.

### 3.2. Case Study

In some cases, drug treatment is not sufficient to ensure the improvement of a patient clinical condition. The monitoring of a patient physical activity allows the development of an individualized therapeutic strategy, complementing the drug treatment. The practice of physical exercise with correct frequency and intensity can help to improve outcomes, specially in the case of cardiac and respiratory diseases.

Recognition and activity intensity measurement systems are intended to infer human movement patterns (e.g., walking, running, sitting, standing) and calculate the intensity with which the activity is being performed by the patient. The inference can be made from data supplied by sensors of different types (e.g., heart rate and accelerometer). The hit rate of inference mechanisms used by the system varies according to several factors, including the accuracy of the sensors and the adopted activity classification technique. By detecting the activity that the patient is performing and the corresponding body stress level, the system can detect situations where the activity performed favors or harms the patient health.

With the recognition and monitoring of the patient activity, health professionals can check if the level of effort used in carrying out the activity is compatible with the physical limits of the patient and make the necessary corrections. These limits vary according to the patient condition, age, weight and other factors.

In order to fill the gap in the described scenario, a context-aware application named Mobile Human Activity Recognition System (MHARS) [69,70] was developed. MHARS is a mobile monitoring system that performs activities recognition, calculates its intensity level, and correlates these data with other context information for inferring relevant situations related to the patient health status. The system was developed by the Laboratory of Intelligent Distributed Systems of the Federal University of Maranhão in partnership with the Center for Research in Nephrology of the UFMA Hospital (HUUFMA).

MHARS correlates the inferred activity and intensity with other contextual information (e.g., the patient disease and body temperature, environmental temperature and height relative to sea level, etc) to detect specific situations related to the patient health status. A decision-making mechanism defines an action plan to be executed when relevant health situations are detected. The actions to be performed are represented using ECA rules (Event-Condition-Action). The system can, for example, issue a warning to a patient with a chronic cardiovascular disease (or to his/her physician) that is running if the intensity level of his/her activity is above the prescribed one.

The general requirements of the system were defined through interviews with health professionals. The main requirements are:

- Interaction with Heterogeneous Physical Sensors: the system must be capable of interacting with different types of sensors (e.g., portable, wearable or embedded in intelligent environments) in order to obtain different types of context data about the patient (e.g., physiological, movement and location) and the environment in which it is located (e.g., light, temperature, air quality);
- Activity recognition: the system must be able to determine the activity performed by the patient in real time, based on the grouping, sorting and processing of the data collected in real time;
- Intensity measurement: The system should be able to measure the intensity of the performed activities, as well as to determine if the intensity is low, high or moderate;
- Situational inference: the system must be able to infer the patient’s state (e.g., patient with atrial fibrillation running with moderate intensity at 1800 m altitude at 3:00 p.m.) by specifying rules whose execution infers different situations based on an analysis that combines data from the sensors, activity and intensity, taking into account the specific treatment of the patient;
- Decision making and execution of actions: the system should be able to perform actions predefined by health professionals for each type of detected situation (e.g., send an emergency notification to health professionals);
- Mobility support: the system must provide mobility to the users, allowing patients and health professionals to use the system to recognize activities in a flexible way and in different situations;
- Local and remote data distribution: the system must be capable of distributing data and context for both the application running on the patient’s mobile device and the applications running on the healthcare providers devices.

To better detect and respond to different situations, MHARS needs to calculate various parameters. The requirements of MHARS regarding the quality of the information are:

- Accuracy: The accuracy rate in activities recognition, intensity measurement, and status inference depends on how the collected context data reflects the reality in which the patient is;
- Available attributes and completeness: the efficiency and success rate of activity recognition mechanisms and situation inference are greater when all the attributes of the collected information are available;
- Sensor location: in emergencies, it may be necessary to inform the location of the patient to a response team, doctors, caregivers or family members.

Finnaly, the MHARS requirements for quality of service are:

- Delivery reliability: the data processed by the mobile device (activity, intensity, situation) must be delivered to the remote server running in a cloud infrastructure. Since mobility requires the use of wireless networks, which are more prone to failures in the transmission, it is important that the system be able to retransmit the data if delivery is not confirmed;
- Refresh Rate: the system must be able to adjust the frequency used for detecting activities and situations, allowing patient monitoring in real-time or only occasionally as needed;
- History: the system must be able to store detected activities, inferred situations and other patient data so that they can be available to the health professionals when necessary.
- Lifespan: the system should provide the means for health professionals to determine the context information expiration time. Therefore, out-of-date data must be removed from the application history.

Those system requirements have contributed to define the M-Hub/CDDL requirements, as it is described in the following section. The implementation aspects related to MHARS are described in details in Section 4.

### 3.3. M-Hub/CDDL Requirements

The following are the main requirements of context aware applications that are served by the proposed middleware.

- Interaction with Heterogeneous Physical Sensors: the middleware must provide mechanisms for interacting with a broad range of heterogeneous physical sensors. It must also allow the opportunistic discovery and ad-hoc interaction with sensors using short-range wireless communication technologies such as *Classic Bluetooth* and *BLE*. The support for wireless technologies must be extensible, allowing easy integration of new technologies as needed.
- Local and Remote Data Distribution: it must provide mechanisms of data distribution, in order to allow information sharing between context producers and consumers in a flexible way. The middleware must support both local distribution (producers and consumers running on the same device) as well as remote distribution (producers and consumers running on different devices).
- Discovery and Registration of Distributed Services: the middleware must provide mechanisms to describe the characteristics of the available context services (at least, publisher id, service name, last read value, and averages of QoC attributes) provided by smart objects so they can be registered in a local and/or in a global directory service. The later should run in a cloud/cluster infrastructure and maintain a register of all discovered services provided by a group of CDDL publishers. Context-aware applications (subscribers) should discover the services available by performing queries on those directories. The query should use an expressive language that allows the developer not only to describe the application demands for context information services but also the required QoC that they must provide. CDDL clients (publishers and subscribers) connected to the same broker are automatically part of the same domain (the CDDL domain).
- Uniform and Location-Independent Programming Model: indicates that the programming model (interfaces, components) used for development of applications that consume local data is the same one adopted for applications that consume remote data. That is, the developer does not have to change the way of programming if the data origin changes.
- Context Information Quality, Monitoring, and Provisioning: the middleware must provide mechanisms that allow the context information to be enriched with attributes that describe its quality (e.g., accuracy, source location, measurement time, expiration time, available attributes and completeness) and provide methods to calculate them dynamically. The mechanisms for registering and discovering services should take QoI into account. The local and/or global directory service must be aware of the provided QoI. The context query language should allow the specification of the consumer QoI requirements for selecting appropriate context services that match the specified criteria. In addition, the middleware must provide monitoring mechanisms that allow consumer applications to be notified when the QoC changes significantly, allowing the application to react to those events.
- Distribution Quality Monitoring and Provisioning: this requirement means that producers and consumers can independently define quality of data distribution service policies. These policies express a set of configurations that determine how CDDL handles a broad set of non-functional properties (e.g., delivery reliability, deadline, refresh rate, latency budget, history, lifespan). The service registration and discovery mechanisms should also take into account the need to store and query information about the quality of the distribution service. For some QoS policies, monitoring mechanisms should be provided to ensure that publishers and subscribers are notified when the required quality is no longer met.
- Reliable Data Delivery in Mobility Scenarios: In order to also handle the distribution of context information in unrestricted mobility scenarios, the middleware must provide mechanisms that minimize data loss, in the presence of weak or intermittent connectivity of local gateways and/or consumer applications with the Internet.

The following subsection describes the middleware architecture highlighting each component functionality.

### 3.4. M-Hub/CDDL Architecture

**Figure 2:**
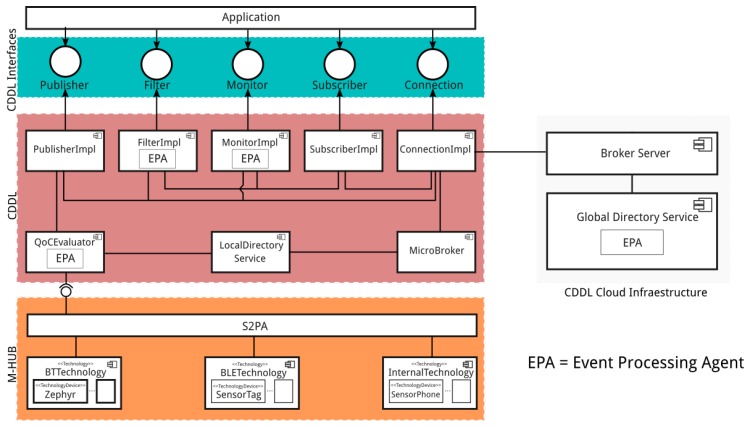
M-Hub/CDDL architecture.

The mechanisms presented in the previous section are implemented by different M-Hub/CDDL components, as can be seen in Figure 2. The figure shows the M-Hub components (Short-Range Sensor Presence and Actuation (S2PA), Bluetooth Classic Technology (BT), Bluetooth Low Energy (BLE) Technology, Internal Technology), CDDL components (QoC Evaluator, Local Directory Service, Publisher, Subscriber, Monitor, Filter, Connection and Micro Broker) and Cloud/Cluster components (Server Broker and Global Directory Service). In the following subsections, the components of each layer are described in detail. Although they can be used in isolation, the architecture presented shows how they can be interconnected.

#### 3.4.1. M-Hub Components

M-Hub is responsible for discovering and interacting with opportunistic sensors as well as monitoring the availability status of each device. This layer provides a generic interfaces that standardizes the way middleware discovery and interacts with heterogeneous devices and technologies. The idea is that M-Hub/CDDL programmers use this interfaces to implement specific adapters/wrappers for each sensor type. The adapters/wrappers encapsulate the sensor driver and expose the services implemented by the device to the upper layers of the middleware. The sensor driver is always supplied by the sensor manufacturer and contains the native software/code required for the interpretation/conversion of the sensor data packets. M-Hub/CDDL programmers can share sensor adapters/wrappers implemented in accordance with these standard interfaces defined by the proposed middleware through Web software repositories. In this way, the M-Hub/CDDL programmers form a community for sharing sensor adapters/wrappers implementations, speeding up the development process. M-Hub is able to download sensors adapters/wrappers from these repositories and load them dynamically. This ability is fundamental for opportunistic interaction with sensors in IoMT scenarios, in which the gateway generally does not know all the sensors it may encounter along the way. Other requirements implemented by the M-hub layer are: data transcoding to a standard format; data caching to optimize data transmission for cloud/cluster processing; in-network (pre)processing of context data through Complex Event Processing rules (details about the use of CEP are in Section 3.7.1) or Java code that can be dynamically downloaded and instantiated; and adaptive power management mechanisms.

A full description of M-Hub is provided in [4,57,62]. In this paper, we highlight the main M-Hub components related to the discovery of nearby smart objects services and context data acquisition. This components are:

- S2PA: component responsible for managing the discovery, connection, and interaction with smart objects. M-Hub standardizes the sensor adapters/wrappers implementation through three main interfaces provided by S2PA: Technology, Technology Device and Technology Listener. S2PA APIs were designed to provide generic methods for short-range communication between the M-Hub and smart objects, that can be directly mapped to the specific capabilities of the underlying short-range wireless communication technologies (WPANs). Hence, for each supported WPAN was introduzed into the M-Hub the component that implements the methods defined by the S2PA APIs. The Technology Interface posses a unique ID that is defined at programming time to identify each technology (e.g., Bluetooth Classic, BLE, ANT+, etc.). That interface has the required methods to handle different short-range protocols used for the interaction with sensors. Some of the main methods defined by the Technology interface are the followings: boolean exists(), method that verifies if the device provides support for the technology; readSensorValue() and writeSensorValue(), methods that request a read or a write of a sensor, respectively. There is an attribute called serviceName (a String), which represents the sensor service (e.g., “Temperature”, “Humidity”, “Accelerometer”). From the M-Hub point of view, each device adapter/wrapper (e.g., Zephyr Bioharness) is a class that implements the Technology Device interface. All the context information and QoC attributes that a sensor adapter/wrapper can provide are send to the Technology Listener, that takes care of sending the data retrieved from the different devices to the CDDL layer, responsible publishing the context data. S2PA also manages the device’s reachability, notifying eventual disconnections that can occur due to several reasons, such as the movement of smart objects or the gateway, considering the unrestricted mobility of IoMT. Once started, the S2PA service periodically searches for available nearby devices supporting the enabled communication technologies. If there are adapters/wrappers compatible with the discovered devices, S2PA will connect to them and start receiving their data (in case of sensors) or wait for remote commands to be sent to the device (for actuators). Whenever a new smart object is discovered, S2PA generates an event for each service provided by the smart object notifying its availability. Local Directory Service (see Section 3.4.2) receives those notifications, registering the discovered services. In addition, S2PA monitors the connection established with the smart object in order to be able to notify the Local Directory Service whenever there is a disconnection (spontaneously or abruptly) and the connection is not restored within a configurable time window. This time window is intended to prevent sending unavailability notifications in situations where the smart object connects and disconnects to the gateway frequently.
- BT Technology and BLE Technology: components that implement the functionality required for the interaction with devices supporting Bluetooth Classic (e.g., Zephyr Bioharness (https://www.zephyranywhere.com/)) and Bluetooth Low Energy (e.g., Sensor Tag (http://processors.wiki.ti.com/index.php/SensorTag_User_Guide)) respectively, based on the operations defined by the Technology interface.
- Internal Technology: component that implements the functionality required for the interaction with built-in sensors available on the Android device running the M-Hub.

#### 3.4.2. CDDL Components

The CDDL components are:

- QoC Evaluator: receives context data enriched with some QoI metadata from S2PA. One of the responsibilities of the QoC Evaluator is to dynamically compute the value of some QoI parameters, which are not provided during data acquisition since they are not intrinsically related to the sensor hardware. The computed QoI is also added to the context data metadata structure and then forwarded to the Publisher. The QoC Evaluator also periodically calculates the average quality of context provided by each sensor. This average QoC is a relevant information for some client applications, which can use it as a criteria for selecting context services. They are stored by the directory services.
- Publisher: component responsible for publishing data, which may be originated from the S2PA or provided by the application layer. From the point of view of client applications, each instantiated Publisher is a context service provider. In addition to publishing context data enriched with QoI metadata, this component can also be used to publish context queries and responses used in the process of searching for smart objects services. Data delivery to the broker is performed according to a set of QoS policies implemented by this component.
- Local Directory Service: responsible for managing information related to the context service providers running on the device. This information includes the available context data types, the average quality of the information published, and data related to the quality of the distribution service offered by each provider. Local Directory Service is also responsible for publishing the service unavailability notification on the same topic used to publish and receive the context data in order to notify the applications that a service is no longer available.
- Subscriber: component responsible for context data acquisition following a topic approach. In this way, applications subscribe to a given context topic. In addition to receiving context data enriched with QoI metadata, a Subscriber can also be used to receive context queries and responses. Data delivery to the application layer is done according to some QoS policies implemented by this component.
- Micro Broker: modified version of a Broker Server targeting mobile devices running the Android platform. Its function is to intermediate the communication between publishers and subscribers based on the definition of topic. A topic is a hierarchical structured string, which is used for message filtering and routing. By default, this component is configured to only accept local connections. In this way, message exchange is only possible between applications running on the same device. However, the Micro Broker can also be configured to accept remote connections, enabling communication between applications running on different devices.
- Connection: component responsible for managing connections and sessions established by clients CDDL with brokers, whether local or remote. An application can establish multiple connections. Each one of them can be either local or remote. For each established connection, an instance of the Connection component is created for managing the connection.
- Filter: component that filters information based on the content of its attributes, including the QoI metadata. MQTT allows clients to publish and subscribe context information based only on the definition/signature of topics that are managed by the broker. However, it does not provide content-based routing/filtering. Therefore, applications use the topic approach to send and receive data to/from the MQTT broker and locally apply the content-based filtering mechanism, if necessary.
- Monitor: component responsible for analyzing context data streams in order to detect the occurrence of certain events that are of interest to the application. The monitoring mechanism is rule-based, an approach that allows the application to define monitoring events of its interest and implement the actions that must be performed when such events are detected. The monitoring mechanism uses a CEP engine which allows event processing to occur in near real-time. For each monitoring rule, a Listener is provided through which the application is notified in case of an event occurrence. This component can also be used for QoI and QoS monitoring. However, some QoS policies are self-monitored, i.e. the policy itself has a standard monitoring mechanism that notifies the application if the required quality of service is violated.

#### 3.4.3. Cloud/Cluster Components

M-Hub/CDDL architecture also comprise the following components running in the cluster or cloud:

- Broker Server: intermediates the communication between publishers and subscribers connected to the cloud. This component has a greater capacity of data distribution than the Micro Broker, since it runs on a hardware with more powerful computing resources.
- Global Directory Service: component responsible for receiving and storing all records of service providers available in the same CDDL domain. By default, each Local Directory Service publishes events that characterize the discovery or disappearance of context services in topics managed by the server broker (if the CDDL client has a connection to the network). The Global Directory Service is a subscriber of these topics and is able to update its records based on the events it receives. However, each Local Directory Services can disable the sending of information to Global Directory Service.

If an application does not know in advance the topics in which the context information of interest is being published it can discover them dynamically. One way to do this, using CDDL, is by publication of context queries. Local and global directory services are responsible for responding to incoming queries based on the records they store. There are two types of queries: instantaneous and continuous. The instantaneous query returns the available service providers that meet a given criteria at that moment the query was issued. Since communication is asynchronous, the application can define a time-out for receiving the response. The continuous query not only returns the service providers meeting the specified criteria at the time it was issued, but also instantiates the query in the Monitor component. In this way, the middleware continuously evaluates the query as new services providers are discovered, returning to the application the ones the match the specified criteria. Both queries can be canceled at any time by the application. Another way that can be used for applications to discover the available services is through subscription to special topics. In these topics, both the Local Directory Service and the Global Directory Service periodically publish updated lists of registered services.

The CDDL is very flexible in respect to the configuration of how context data flows can be established. A Publisher or Subscriber can be connected to the local Micro Broker running in their same machine or can establish a connection with a Micro Broker running in any CDDL machine. They can also be connected to the Server Broker running in the CDDL cloud/cluster infrastructure. It is also allowed for Publishers and Subscribers to establish multiple connections. If they are connected only to Micro Brokers, queries issued for service discovery will have a local scope. On the other hand, if they are connect to the Server Broker, service discovery will have a global scope. The use of MQTT brokers for both local and global distribution flows allows the development of IoT/IoMT applications that, regardless of data location (local or remote) and the platform they run on (desktop stations or mobile devices), the same programming model and protocol, as well as the same quality of distribution service policies are available to the application programmer. The asynchronous communication is implemented using the Paho MQTT Client Library (https://www.eclipse.org/paho/clients/java/). This library releases M-Hub/CDDL client from having to wait for a confirmation before attempting to send another message. Instead, it receives delivery confirmations in background using listeners/callback functions.

### 3.5. Quality of Information Parameters

The CDDL provides an extensive support for both, QoI and QoS parameters. The available quality of information parameters are:

- Accuracy: represents how close the measured value is to the actual value [8]. Generally, IoT applications work with the accuracy estimated by the sensor. In some cases, sensor defects and/or failures can cause the estimated accuracy value to be significantly distant from the actual value. For mobile devices, S2PA obtains the internal sensors accuracy using the Android sensors API. However, for external sensors (BT and BLE sensors), it is necessary for the sensor driver programmer to use its APIs to obtain the accuracy and inform S2PA. After getting the accuracy S2PA informs CDDL.
- Source Location: is the location where the context information was measured [8]. In M-Hub/CDDL, indicates the approximate position of the context source at the time of measurement. If the smart object has a built-in location hardware, its provided location will be assigned to the gathered data. However, in most cases, smart objects do not have their own location hardware. In this case, the QoC Evaluator will assign to the data the position of the device it runs on. Since short range wireless communication networks are used for interaction with smart objects, the provided location will only be an approximation of the context source location.
- Measurement Time: is the moment at which the context information was measured [8]. In M-Hub/CDDL, indicates the approximate time it which the information was measured at the smart object. If the smart object does not provide this information, then the QoC Evaluator will fill the corresponding metadata with the timestamp of data arrival. Note that in this case the elapsed time between the measurement and the arrival of the data through the WPAN communication is neglected. This usually takes miliseconds. A similar approach is adopted for internal sensors, except for the fact that there is no short network communication, leading to a even better precision.
- Arrival Time: informs the arrival timestamp of the context information at the consumer. The arrival time is entered in the context information by the *Subcriber* component.
- Expiration Time: is the time interval in which the context information can be considered valid for the applications, counting from the measurement time [8]. The expiration time is specified by the producer of the information, and can be considered or not by consumer applications. Upon receiving the information, the consumer can accept the specified vality time or even extend this time according to its own interests.
- Age: is the elapsed time between the measurement time of the information and the current instant [8], calculated as follows:
  (1) $$Age=t_{curr}-t_{mens}$$
  where $t_{curr}$ is the current time and $t_{mens}$ is the measurement time.
  Therefore, age is a very dynamic parameter, which must be recalculated whenever it is required for some decision making.
  In M-Hub/CDDL, the parameter is dynamically calculated based on the difference between the measurement time and the current time. When the producer and the consumer run on different devices, a time synchronization mechanism is required in order to calculate the age of the context information at data receipt as accurately as possible (e.g., ClockSync (https://play.google.com/store/apps/details?id=ru.org.amip.ClockSync&hl) synchronizes device system clock with atomic time from Internet via Network Time Protocol -NTP).
- Measurement Interval: is the minimum separation interval between two sensor measurements [8]. This parameter is calculated dynamically by the QoC Evaluator based on the difference between the measurement times of two consecutive samples of the same context information. Although the sensors on the Android platform may have their frequency set, the actual sampling rate is not always the same as that specified. Therefore, M-Hub/CDDL adopts the strategy of calculating this parameter at runtime.
- Available Attributes: are the attributes that characterize a given information type [8]. For example, location information provided by GPS has three attributes: latitude, longitude, and altitude. Under normal conditions, all context information attributes are available. However, sensor failures can cause incomplete readings.
- Completeness: indicates how complete is the information that the consumer receives. In other words, it is the amount of information that is provided by a context object, and can be calculated based on the ratio of the sum of the weights of the available attributes and the sum of the weights of the total number of attributes that are required by the consumer. The equation for this metric is as follows:
  (2) $$Completeness=\frac{\sum_{j=0}^{m}w_{j}}{\sum_{i=0}^{n}w_{i}}$$
  where *m* is the number of available attributes and *n* is the total number of attributes for the desired information. The weights *w* are necessary due to the fact that each attribute may have a different importance for each consumer. For example, if the location sensor provides only the latitude and longitude attributes, but not the altitude, then the reading completeness is approximately 66.6%. M-Hub/CDDL assumes, by default, the same weight for all attributes. In M-Hub/CDDL, completeness is calculated dynamically by the QoC Evaluator. The lower the completeness value, the more incomplete is the information received.
- Numeric Resolution: indicates the degree of detail of the context information [6]. It is called granularity by [8]. In M-Hub/CDDL, for numerical data, the QoC Evaluator calculates the numeric resolution as the granularity in relation to the number of decimal places of the measured value. It should be noted that having a sensor that performs temperature readings with two decimal places, such as 20.01 ${}^{\circ }$C for example does not necessarily imply that it has a higher accuracy than another one which measures with only one, 19.0 ${}^{\circ }$C for instance.

### 3.6. Quality of the Distribution Service Policies

In the M-Hub/CDDL, the available quality of the distribution service policies are:

- Deadline: Publishers and subscribers use this policy to specify the maximum time they are willing to wait to send and receive, respectively, at least one message. To time the deadline, this policy uses a local Java TimerTask. This component notifies the application through a Listener when the specified maximum wait time expires. The maximum wait time is set in milliseconds, with the default value of this parameter being “disabled” (deadline equal to zero). In this case, the application will not receive any notification. Publishers and subscribers define the maximum waiting time independently of each other, meaning there is no negotiation between them.
- Refresh Rate: Publishers and subscribers use this policy to specify the minimum separation interval between successive messages submissions and receipts, respectively. This policy uses a time-based filter that causes only the last (most recent) message to actually be sent or received. To control the send and receive intervals, this policy uses a local Java TimerTask component. The minimum separation interval is defined in milliseconds, with the default value of this parameter being “disable” (minimum separation interval equal to zero). Publishers and subscribers do not negotiate the minimum separation interval. This way, even if the publisher sends data with a very high frequency, the subscriber can establish a lower reception rate that meets their requirements.
- Latency Budget: This policy is used by publishers and subscribers to set an additional delay in sending and receiving messages, respectively. The definition of an additional delay greater than zero causes the middleware to accumulate the messages produced in the specified interval and send or receive them in a single burst by means of a packing message (a message in which all accumulated messages are inserted). Therefore, if the packager message is lost, any messages entered into it will also be lost. To delay sending and receiving messages this policy uses a local Java TimerTask. The additional delay is set in milliseconds, with the default value of this parameter being “disable” (additional delay equal to zero). In this case, the middleware will not delay sending/receiving messages. Publishers and subscribers define the additional delay independently of each other. Therefore, even if the publisher does not set an additional delay, the subscriber can do so in order to receive the messages in a grouped fashion at regular intervals.
- History: This policy is used by publishers and subscribers to set the amount of message that can be stored in their respective history. This policy can be configured in two ways: *keep all* and *keep last*. In the *keep all* mode all messages are stored in the history (up to resource limits). In *keep last* mode only the last *n* messages are stored in history (*n* needs to be defined by the application). The default setting for this policy is *keep last*, where the size of the history is equal to 1. Publishers and subscribers define the type and size of the history independently of each other, that is, there is no negotiation between them.
- Destination Order: This policy determines how messages are stored in the history of publishers and subscribers. In the case of the publisher, the messages are always sorted by the measurement time (the arrival time of the data at the gateway, if the measurement time is not informed by the sensor). In the case of the subscriber, the messages can be sorted by the measurement time, publication time or message reception time at the subscriber. The default ordering of the subscriber is the message reception time. If the subscriber sets the order by the measurement time or the publication time, it is necessary to install clock synchronization software, since the middleware does not provide this type of functionality.
- Lifespan: This policy is used by publishers and subscribers to control the lifespan of messages. This policy uses a local Java TimerTask that removes from the history messages whose expiration time has passed. The default value of lifespan is “disable” (lifespan equal to zero), indicating that the messages do not have a given lifespan and therefore will not be removed from the history (unless replaced by more recent messages in situations where the history is set to “keep last”). This policy is flexible in relation to the agent of the lifespan definition. By default, the lifespan agent is the publisher (in an analogy with the real world, it is always the product manufacturer that specifies the expiration time). In this case, the message will be removed from both the publisher’s history and the subscriber’s history. It is important to note that if the message reaches the subscriber with the lifespan expired, it is not entered in the history. However, there is an alternate setting, in which the subscriber ignores the lifespan specified by the publisher and sets a different lifespan, which may be larger or smaller than that set by the publisher (in an analogy with the real world the consumer sometimes discards products before the expiration date and sometimes uses products even after the expiration date). This setting is intended to allow the subscriber not to be forced to accept the lifespan imposed by the publisher. There is no negotiation process between publisher and subscriber regarding lifespan.
- Retention: the publisher uses this policy to signal that the broker should retain the last (and only the last) post published on the topic. For the publisher, the default value is “disable”, indicating that the broker should not retain the messages. Retention allows late subscribers, i.e., subscribers who were offline during message posting, to receive the retained message as soon as they (re)connect to the broker. Thus, the subscriber is not required to wait until the next publication to receive an update. However, the client application will only receive retained messages if the subscriber has the retention policy enabled. The default value of this policy for the subscriber is also “disable”, indicating that the subscriber will not pass on any retained messages to the client application. There is no negotiation between publishers and subscribers regarding message retention.
- Vivacity: this policy allows clients to be notified of connection failures on other clients in the same CDDL domain. In order for client B to receive client A fault notifications, client A must register with the broker a *Last Will Testament (LWT)* message at the time of connection. If the client A connection with broker fails, then the broker will publish the LWT message in the topic “Vivacity”. Therefore, client B will only receive the notification if it subscribes to this topic. Receipt of notifications can be filtered based on client ID. In this way, client B may indicate that it wants to be notified only of client A faults, instead of receiving failure notifications from other clients. The default policy value for the client is “disable”, indicating that it does not want to register LWT messages with the broker at the time of the connection. There is no negotiation between publishers and subscribers regarding this policy.
- Reliability: sets the reliability level to be used when delivering messages. There are three possible values: 0 (at most once), 1 (at least once), and 2 (exactly once). At level 0, the best effort policy is used. The message delivery is not confirmed by the receiver. It is not stored by the sender and can be lost in the event of a delivery failure. This is the fastest transfer mode. At level 1, the receiver must send an acknowledgment to the sender. If the sender does not receive the acknowledgment, the message will be sent again with the DUP flag set until the delivery confirmation is received. As a result, the receiver may receive and process the same message several times. The message must be stored locally on the sender buffer until the acknowledgment has been received. If the receiver is a broker, it sends the message to the subscribers. If the receiver is a client, the message will be delivered to the subscriber application. At level 2, at least two pairs of transmissions are used between the sender and receiver before the message is deleted from the sender. In the first pair of transmissions, the sender transmits the message and gets an acknowledgment from the receiver notifying that it has stored the message. If the sender does not receive the acknowledgment, the message is sent again with a DUP flag set, and this is periodically performed until an acknowledgment is received. In the second pair of transmissions, the sender tells the receiver that it has received the acknowledgment by sending it a “PUBREL” message. If the sender does not receive an acknowledgment of the “PUBREL” message, the “PUBREL” message is sent again until an acknowledgment is received. The sender deletes the message from its buffer when it receives the acknowledgment of the “PUBREL” message. The receiver can process the message in the first or second phases, provided that it does not reprocess the message. If the receiver is a broker, it publishes the message to the subscribers. If the receiver is a client, it delivers the message to the subscriber application. This is the slowest and most expensive transmission mode. The default reliability value is level 0. There is no negotiation process between publishers and subscribers regarding this policy. However, the subscriber’s reliability requirements will only be met if the publisher uses a reliability level greater than or equal to that required by the subscriber. For example, if the subscriber requires reliability level 1, then the publisher needs to use reliability level 1 or 2.
- Session: defines whether the session held between the client and the broker is persisted or not. A persistent session is one in which the broker stores the customer’s identifier and the topics signed by him, preventing data from having to be re-informed in the case of reconnection. During a temporary disconnect, the broker stores messages posted on topics that were of interest to the subscriber in a buffer, but only those that require delivery confirmation. When the client reconnects, the broker will attempt to send (or resend) the persistent messages, obeying the order of receipt. The default value for this policy is “not persistent”, that is, the broker does not save the session between the subscriber and itself and, therefore, does not attempt to resend persisted messages when the connection is restored.

Note that in order to minimize the message losses in mobility scenarios caused by intermittent connectivity, publishers and subscribers must enable both delivery reliability and persistent session. On the one hand, enabling reliability without using a persistent session means that the subscriber will lose messages that were posted while off-line, as those messages will not be persisted by the broker if the client fails. On the other hand, if the subscriber only enables a persistent session, this will imply that none of the messages will be persisted in the event of a connection failure, because the persistence mechanism only applies to those messages for which delivery confirmation is required.

### 3.7. Implementation Aspects

M-Hub/CDDL executables and documentation are available for download on the middleware webpage (http://www.lsdi.ufma.br/projetos/cddl/doku.php), where detailed instructions and examples can be found about how the various middleware mechanisms should be configured and used to develop applications that have management and quality of context requirements. This section presents only a few details about the operation and implementation of some of the mechanisms that have to do with QoC: evaluation, service discovery, monitoring and filtering. Since these mechanisms are based on complex event processing (CEP), a brief explanation of this technology precedes the description of such mechanisms.

In addition to the components mentioned in this section, the S2PA component plays a key role in acquiring context information from external sensors. The implementation aspects of this component and the interfaces used by the developers when using it are detailed in the works referring to the M-Hub, nominally Rios et al. [4] and Vasconcelos et al. [57].

#### 3.7.1. Complex Events Processing

Complex Events Processing [71] is a technology that allows correlating continuous input events and patterns of interest (e.g., filtering, aggregation, summarization, differentiation, enrichment) where processing results can be other complex events, that is, events that are derived from the input events. CEP is a low latency (in terms of processing) method of tracking and analyzing of data flows. This method combines data from multiple sources to infer events or patterns that suggest more complicated circumstances [51]. CEP is used to process data flows in near real-time as well as to produce results without delays, even in cases where the flow of events is large. In a reversal compared to traditional database management systems, where a query is performed on stored data, CEP performs data in a stored query. Near real-time is a level of computer responsiveness that user senses as sufficiently immediate (in the order of milliseconds), that is, there is no visible delay for the  user.

One of the CEP advantages is the possibility of using declarative languages to define processing queries, known as Event Processing Languages (EPLs) [71]. EPLs allow one to express rich conditions and correlations between events as well as time window concepts, thereby minimizing the development effort required to configure systems that can react to complex situations [72]. Due to the expressiveness of EPLs, complex event-detection scenarios that were previously difficult to implement using other technologies can now be specified with few lines of code, favoring the reuse of solutions. Support for running event flow queries, written in EPL, is provided by CEP engines. There are several CEP engines, one of the best known being the *Esper CEP* (http://www.espertech.com/esper/) [72], an open source engine written in Java. The EPL Esper is a rich, continuous query specification language, based on Structured Query Language (SQL), but also includes several language-specific constructs for processing real-time event flows, such as time windows [51]. M-Hub/CDDL uses Esper Engine in the CDDL desktop/web version and Asper [73], a modified version of Esper Engine for Android devices, in the CDDL mobile version.

#### 3.7.2. QoC Evaluation

As mentioned in Section 3.4, in addition to automatically computing some of the QoC parameters, when not provided by the producer/sensor, QoC Evaluator also calculates an average value of QoC within a given time window. This average serves as an indicator of the QoC offered by the producer and can be used as a reference value/metric that helps context consumers select the service provider best suited to their context and QoC requirements. For this reason, average QoC values, as well as all other information that characterizes the service provider, are sent to the service directories so that they can be subsequently queried by consumer applications.

QOC Evaluator is implemented as a Java thread in the desktop/web version of CDDL and as a Android service that runs in the background in the mobile version, and uses CEP to analyze sensor data publication flow and thus detect new types of context as well as apply aggregation functions that calculate the average quality of context of the information. The events that result from this processing generate a new stream of events that are processed by the *QoC Evaluator* itself. This new flow is used by the Local Directory Service to respond to service queries published by applications.

Listing 1 shows an example of the EPL Esper rule used to compute the average QoC. This rule captures events of type *SensorDataMessage*, which corresponds to the message type published by S2PA. This message contains the context data and its QoC attributes, within a configurable time window. The result of processing these events is the creation of new events of type *ServiceInformationMessage*, which corresponds to the type of message that contains the description of the available services. These new events are sent to the local and global directories, which update their records based on this information.

Listing 1 **EPL used to compute the average QoC.**


insert into ServiceInformationMessage (
  publisherID, serviceName, accuracy, measurementTime, availableAttributes,
  sourceLocationLatitude, sourceLocationLongitude, sourceLocationAltitude,
  measurementInterval, numericalResolution, age
) select publisherID, serviceName, avg(accuracy), avg(measurementTime),
  avg(availableAttributes), avg(sourceLocationLatitude), avg(sourceLocationLongitude),
  avg(sourceLocationAltitude), avg(measurementInterval), avg(numericalResolution), avg(age)
  from SensorDataMessage.win:time(TIME_WINDOW)
  group by publisherID, serviceName

SensorDataMessage is a specific type of message, which inherits from the Message class. A simple example of how this type of message is published is shown in Listing 2:

Listing 2 **Publication context data with QoC example.**

// creates the message
SensorDataMessage msg = new SensorDataMessage();
// fill the message properties
msg.setServiceName(‘‘TEMPERATURE’’);
msg.setServiceValue(38); // the type can be any Java type (String, Integer, Object, etc.)
msg.setAccuracy(0.025); //or any other QoC parameter
// obtaning references to default publisher and publishes the message
Publisher publisher = MHubCDDL.getInstance().getDefaultPublisher;
publisher.publish(msg);

#### 3.7.3. QoC-Based Service Discovery

As mentioned in Section 3.4, consumer applications discover the available service, as well as the QoC offered by them, through instant and continuous queries. These queries are also written in EPL Esper and allow the application to specify criteria about the type of context and level of QoC required. Listing 3 shows an example of how the application publishes queries using the CDDL. In this example, the application invokes the query(QueryType queryType, String epl) method of the *Publisher* component. This method receives the EPL query to be published as a parameter. The specified query searches for all location services whose accuracy is less than 5 m. The example references messages of type ServiceInformationMessage in the EPL clause since this is the data structure that contains the services description. In this example, the query is continuous, therefore the consumer would receive all available services compatible with the query as they are discovered.

Listing 3 **Example of query written in EPL Esper.**

// obtaning references to default publisher and subscriber
MHubCDDL mhubcddl = MHubCDDL.getInstance();
publisher = mhubcddl.getDefaultPublisher();
subscriber = mhubcddl.getDefaultSubscriber();
// query for location of the publisher johndoe@example.com with accuracy less than 5;
String query = ‘‘serviceName = ’LOCATION’ and publisherID = ’johndoe@example.com’
  and accuracy < 5’’;
// returnCode can be used to cancel the continuous query
int returnCode = publisher.query(QueryDestiny.LOCAL, QueryType.CONTINUOUS, query);
// creates the listener to receive subscriptions
subscriber.setSubscriberListener(new ISubscriberListener() {
  @Override
  public void onMessageArrived(Message message) {
    // handles query responses
    if (message instanceof QueryResponseMessage) {
    QueryResponseMessage response = (QueryResponseMessage) message;
      // is the query response about location of johndoe@example.com?
      if (message.returnCode == returnCode) {
        // obtain the list of services from response
        List<ServiceInformationMessage> services =
          response.getServiceInformationMessageList();
        // loop from all services from response and subscribe them
        for(ServiceInformationMessage info : services) {
          subscriber.subscribeServiceTopic(info.getTopic())
        }
      }
     return;
    }
    // handles subscribed messages
    if (message instanceof SensorDataMessage) {
      SensorDataMessage sensorDataMessage = (SensorDataMessage) message;
      // simulates showing message on UI.
      showMessageOnUI(message);
      // one can get the attributes of the message like:
      // message.getPublisherID();
      // message.getServiceName();
      // message.getServiceValue();
      // message.getAccuracy();
      // message.getSourceLocation();
      return;
    }
  }
  // if, in any moment, the application wants to cancel the query above
  // publisher.cancelQuery(returnCode)
});

Queries performed by consumer applications are received by local and global service directories (LocalDIrectoryService and GlobalDirectoryService respectively) that use CEP engines for processing. The LocalDirectoryService component is implemented as an Android service while GlobalDirectoryService is a Java process running in the cloud.

#### 3.7.4. QoC-Based Event Monitoring

Monitoring of variations in sensor measurements and QoC itself is also based on the specification of EPL rules. However, unlike the evaluation and service discovery mechanisms, which implement the CEP rules on producer-side data flows only, the monitoring rules can be applied to both sending and receiving flows, that is, both the publisher and the subscriber can detect context and QoC monitoring. As mentioned in Section 3.4, the component responsible for monitoring is the Monitor. This component is implemented as a Java class that is instantiated by publishers and subscribers when needed.

To exemplify monitoring of the QoC variation, let us consider the following example: an application wants to be warned whenever the accuracy of the temperature of a particular publisher changes. In this case, the following CEP rule could be passed to the Monitor: select * from pattern [every A=SensorDataMessage -> B=SensorDataMessage and A.accuracy <> B.accuracy] where A.serviceName = ’TEMPERATURE’ and A.publisherID = ’bertodetacio’.

Therefore, code in Listing 4 can be used in the above scenario:

Listing 4 **Example of using the M-Hub/CDDL monitoring engine.**

// first, define a monitor listener
IMonitorListener monitorListener = new IMonitorListener() {
  @Override
  public void onEvent(final Message message) {
    // the monitoring event just happened.
    // do something about it
    doSomething(message);
};
 
// monitoring rule
String cepRule = ‘‘select * from pattern
  [every A=SensorDataMessage -> B=SensorDataMessage and A.accuracy <> B.accuracy]
  where A.serviceName = ’TEMPERATURE’ and A.publisherID = ’johndoe@example.com’’’;
 
// ruleId can be used to remove the query (see comment below)
int ruleId = subscriber.getMonitor().addRule(cepRule, monitorListener);
 
// cancel the monitoring using the statement bellow
// subscriber.getMonitor().removeRule(ruleId);

An inherent aspect of monitoring is that it can be enabled and disabled according to the interests of the application. The example showed how to remove a monitoring rule dynamically.

#### 3.7.5. QoC-Based Event Filtering

Event filtering is also based on the specification of EPL rules. As with monitoring, filtering rules can be applied to both sending and receiving flows, that is, both the publisher and the subscriber can filter out context information, including QoC-based filtering. As mentioned in Section 3.4, the component responsible for filtering is the Filter. Like Monitor component, Filter is implemented as a Java class that is instantiated by publishers and subscribers on demand.

To exemplify QoC-based filtering, let us consider the following example: an application that subscribes to receive temperature data from multiple publishers and at a certain point wishes to receive only data with accuracy greater than 0.9. In this case, the code shown in Listing 5 can be used:

Listing 5 **Example of using the M-Hub/CDDL filtering engine.**

// subscribe to temperature of any publisher
subscriber.subscribeSensorDataTopicByServiceName(‘‘TEMPERATURE’’);
// the application will only receive context information with accuracy equal to 0.9
subscriber.setFilter(‘‘select * from SensorDataMessage where accuracy > 0.9’’);
// disable filtering and receive all information
subscriber.clearFilter();

Filtering can be canceled at any time through the clearFilter() method. From this moment on, all subscribed information is received normally.

#### 3.7.6. QoS Configuration

From the application point of view, each QoS policy is represented by an class that contains specific methods for configuring QoS parameters. Except for the Session policy, all policies inherit from the AbstractQoS class, which can be used to implement new policies. Once defined, the Publisher and Subscriber interfaces are used to change publisher and subscriber policies, respectively.

Listing 6 shows an example of how to configure the Reliability policy. The configuration of the other policies is done in a similar way.

Listing 6 **Reliability configuration example.**

// informs the customer ID that will be used by all connections
MHubCDDL.getInstance().setClientId(‘‘bertodetacio@gmail.com’’);
 
// obtains the connections factory instance
ConnectionFactory connectionFactory = ConnectionFactory.getInstance();
 
// get an instance of the connection
Connection connection = connectionFactory.createConnection();
 
// activates the intermediate buffer
connection.setEnableIntermediateBuffer(true);
 
// instantiates the reliability policy
ReliabilityQoS reliabilityQoS = new ReliabilityQoS();
 
//configures reliability at level 1
reliabilityQoS.setKind(ReliabilityQoS.AT_MOST_ONCE);
 
// gets a default publisher instance
Publisher publisher = DefaultPublisher.getInstance();
 
// changes publisher reliability policy
publisher.setReliabilityQoS(reliabilityQoS);
 
// adds a connection to the publisher
publisher.addConnection(connection);
 
//connects
connection.connect();

## 4. Implementing an Application with QoC Requirements

Mobile Human Activity Recognition System (MHARS) [69,70] is a mobile monitoring system that performs activities recognition, calculates its intensity level, and correlates these data with other context information for inferring relevant situations related to the patient health status. The system runs on Android mobile devices and uses a cloud computing infrastructure to store and retrieve the information inferred from the patients. MHARS can recognize the following activities: walking, running, jumping, standing, lying down, up and down the stairs. Acceleration data and machine learning algorithms are used for activity recognition. The heart rate is used to calculate the activity intensity. This section describes how MHARS was implemented using the M-Hub/CDDL and discusses the advantages of using the middleware proposed in the development of the case study described in Section 3.2.

### 4.1. System Architecture and Key Features

The first version of MHARS was developed using the original version of M-Hub (1.0) as the acquisition layer, and the Scalable Data Distribution Layer (SDDL) as the distribution layer. In this version, the implementation of QoC requirements was the responsibility of the programmer, since both M-Hub and SDDL did not support QoC at the time. However, MHARS version 2.0 was developed using the middleware approach proposed in this work, which uses a modified version of the M-Hub and replaces the SDDL with the Context Data Distribution Layer CDDL (see Section 3), which offers QoC support.

**Figure 3:**
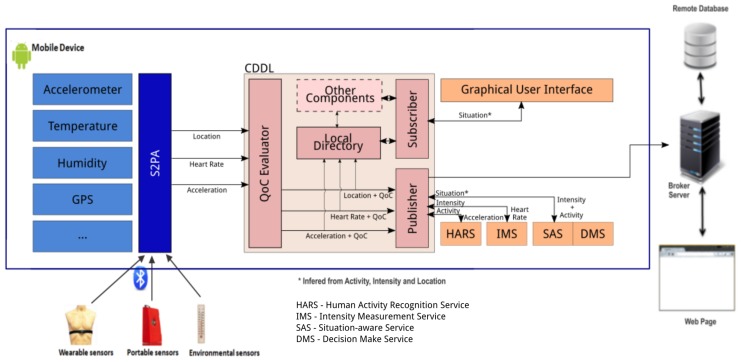
MHARS architecture using M-Hub/CDDL components.

Figure 3 presents the MHARS 2.0 architecture. It shows the M-Hub/CDDL components and the specific components that implement business system logic. The components are organized into two subsystems: (i) the subsystem of data acquisition, activity recognition, intensity measurement, status inference and decision making, which runs on mobile devices; and (ii) the subsystem of storage and data visualization, which runs in the cloud.

The S2PA is the middleware layer component responsible for interaction with external sensors (using a WPAN) and the patient’s internal sensors smartphone (using the Android API) used as gateway AAL. The S2PA receives data from the heart rate and acceleration in three axes (X, Y, Z), collected by a wearable sensor device called Zephyr BioHarness (http://zephyranywhere.com/products/BioHarness-3/). To make the activities recognition engine most efficient, the system also receives acceleration data collected by the mobile device itself, plus the location (latitude, longitude, and altitude). The QoC Evaluator calculates the QoC parameters required by MHARS and inserts them as meta-data in each context information data sample.

The Publisher publishes the context data to be processed by Human Activity Recognition Service (HARS), Intensity Measurement Service (IMS), Decision Make Service (DMS) and Situation-aware Service (SAS) components on the mobile device. The local application signs the topics in which the data is published, using the Subscriber component. Once all topics are defined at compile time, the local application does not need to consult the Local Directory Service. By default, the local application receives the acceleration data at a rate of 128 samples every 2.58 s. This time window and their number of samples are considered sufficient for the activities of detection performed repeatedly [74]. The default refresh rate for receiving heart rate and location data is 1 sample every 2.58 s. All data are correlated by the measuring time.

The HARS is the application layer component that is responsible for conducting the recognition of the activity being made by the user based on the acceleration data. This component performs preprocessing steps (conversion, filtering and refining) that put the data into an input format compatible with the activities classifier.

The activities classification is performed by machine learning algorithms to recognize the actual activity patterns based on previously processed values. It can use several machine learning algorithms, and most of them are provided by a library called Waikato Environment for Knowledge Analysis (WEKA) [50] that is specific to pattern recognition problems. Depending on the algorithm used, the accuracy rate can approach 83%. Activity recognition systems with accuracy rate exceeding 70% are considered satisfactory [75].

The IMS is responsible for measuring the activity intensity inferred by HARS. This calculation takes into account the current heart rate and the maximum heart rate that can be achieved by the patient. From this information, the IMS can determine if the intensity of carrying out the activity is low, moderate or severe.

The SAS is responsible for inferring the different pre-defined situations in which the patient can be during the course of an activity. The situation inference can consider various information such as the detected activity, the measured intensity, the obtained location, chronic condition of the patient, and other context data. The SAS has an intrinsic relationship with the DMS, the component responsible for defining and implementing the actions necessary to respond to an inferred situation. The decision-making is based on the execution of ECA rules (Event-Condition-Action).

The high-level context information representing the activity, its intensity, and inferred situation, are published by the application in the CDDL cloud. A web application implemented as a CDDL web client can be used by a medical staff, for example, to monitoring the patient and take actions when appropriate. By default, the data are published as soon as available, in order to minimize the delay. Regarding the location, the Filter component prevents activities and situations being published in certain geographic coordinates defined by the local application. At the cloud, an application signs the topics where the high-level context information are published. The refresh rate can be set dynamically and depends on the minimum interval with which application would like to be notified about the patient activities and situations. By default, the refresh rate is 2.58 s. The application can specify the size of the history and the time by which the received data samples are still available.

### 4.2. Discussion on the Case Study

MHARS is an IoT application with a certain degree of complexity because, in addition to requirements such as activity recognition, intensity measurement, situation inference, and others that are inherent in its business logic, this application has several context management requirements (acquisition, processing, distribution, and storage), quality of context requirements, both QoI (accuracy, available attributes, completeness) and QoS (reliability, refresh rate, history, expiration time) as well as mobility. In MHARS version 1.0, the implementation of these requirements was the responsibility of the programmer, since both M-Hub and SDDL did not support QoC at the time.

The case study showed that MHARS’ quality of context and management requirements were met very satisfactorily by M-Hub/CDDL. MHARS required the use of both the M-Hub and the use of two types of CDDL client: the mobile version and the web version. Unlike MHARS version 1.0, implemented with the use of SDDL, this time it was not necessary for the programmer to implement such requirements at the application layer and/or middleware because CDDL provided all the QoC support that the application required. Thus, from this particular case study, it can be generalized, to some degree, that M-Hub/CDDL is applicable to the development of a series of IoT applications in the health domain. In addition, considering that applications from other domains, such as transport, commerce, and industry, for example, may have similar context management and QoC requirements, it can also be deduced that M-Hub/CDDL can be used in the development of IoT applications in various domains. Although it was not possible to apply all the concepts and mechanisms present in the M-Hub/CDDL in MHARS, this case study showed that they are valid, that is, they can be applied in the resolution of the problems to which the middleware is intended, fulfilling so the objective of M-Hub/CDDL, which is to facilitate the development of context-aware IoT applications, especially those that have multiple QoC requirements.

## 5. Evaluation and Results

A complete evaluation of the M-Hub/CDDL, due to its wide range of functionalities that aim to support the development of context aware applications with QoC requirements, could take into account several aspects. We present next the evaluation of the following aspects related to the performance of the distribution and monitoring services of the M-Hub/CDDL: (i) evaluation of the delivery time of the context data to the consumer, considering different levels of reliability and network configurations; (ii) the efficiency of delivery reliability policies implemented by the distribution service, considering various scenarios of intermittent connectivity; (iii) the performance of the quality of context monitoring mechanism, in relation to the QoC variation detection time, considering different data publication frequencies and (iv) evaluation of memory and battery consumption.

### 5.1. Evaluation of the Context Information Delivery Time to the Consumer

Before arriving at the consumer, the context data passes through different stages/components of the middleware: (1) the data is collected by the S2PA Service, responsible for the connection and interaction with the sensors; (2) the data is processed by the QoC Evaluator, responsible for evaluating the QoI and generating the Local Directory Service records; (3) the data is enriched with QoC and sent to Publisher so that it is published in accordance with the producer QoS settings; (4) the context data is sent to one or more brokers via Connection Service; (5) each broker that receives the context information, be it local or remote, sends this information to the subscribers registered in the corresponding topic; (6) the context data arrives at Subscriber, where it is handled in accordance with subscriber QoS settings; (7) the information is delivered to the application layer by means of a *listener*, registered by the application itself.

The purpose of this set of experiments is to evaluate the M-Hub/CDDL performance distribution service. In addition, this experiment aims to demonstrate how different configurations of the delivery reliability policy provided by the middleware and various types of networks can influence this performance. To achieve this goal, the following evaluation metrics were defined:

- Communication Time: equals to the sum of the delay imposed by the network communication, or local bus, with the message processing time in the local or remote MQTT *Broker*. Communication time between publisher and subscriber is directly affected by the level of delivery reliability used by publishers and subscribers, as well as by external factors over which the middleware has no control, such as bandwidth, latency, and network packets loss rate.
- Middleware Processing Time: is the sum of the times that the context information takes to transit or be processed by the middleware components in the producer (interval between the arrival of the data at the publisher and sending the message to the MQTT *Broker*) and consumer (interval between the arrival of the message at the subscriber and delivery of the data to the application). The information processing time in the middleware includes marshaling and unmarshaling operations, QoI evaluation, and application of QoS policies according to parameters set by publishers and subscribers.
- Delivery Total Time: it is equivalent to the sum of the communication times between publisher and subscriber and the middleware processing time, that is, the time elapsed between the arrival of the data in the gateway and its effective delivery to the consumer application.

These metrics are natively computed by M-Hub/CDDL, in milliseconds, being made available to the application as metadata.

Communication time is calculated based on the difference between the timestamp of the message when it is sent by the Publisher component and the timestamp of the message when it arrives at the Subscriber component according to the following equation:

(3) CommunicationTime=PublisherTimestamp−SubscriberTimestamp

Delivery total time is calculated based on the timestamp of the information when it arrives in the S2PA component and the timestamp of the message when it is effectively delivered to the consumer application, according to the following equation:

(4) TotalDeliveryTime=S2PATimestamp−ApplicationTimestamp

It should be noted that the arrival time of the information at the Subscriber is not equal to the delivery time of the information for the application. The difference between these times depends on the QoS parameters of the subscriber. For example, if the subscriber uses the *Latency Budget* parameter, then the middleware will impose an additional delay on delivering the message to the application.

Middleware processing time is calculated by subtracting the communication time from the total delivery time, according to the following equation:

(5) ProcessingTime=TotalDeliveryTime−CommunicationTime

The experiment consisted of using a test application that publishes and receives data. Both publisher and subscriber run on the same device and therefore there was no need for clock synchronization to calculate the defined metrics. In each experiment, the publisher sends 1800 messages to the broker, with the data upload rate being approximately 1 Hertz (Hz). The duration of each experiment is approximately 30 min. The size of the *payload* of each message is approximately 1024 bytes.

To support the execution of this application (and consequently of the experiments), the following hardware resources were used:

- Wearable Sensor: used to generate the context data. The Zephyr Bioharness 3 device, which has several sensors, was used. However, the S2PA was set up to collect only heart rate data. The data generation frequency of this sensor is approximately 1 Hz.
- Smartphone: used to run the test application. The smartphone model is a LG-K430F, which has 2 GB of RAM, quad-core 1.2 Gigahertz (GHz) processor, Android 6.0 operating system, Bluetooth and 802.11 Wifi.
- Notebook: used to run the test application monitoring tool (Android Device Monitor) and to run a server broker in experiments using LAN. It was used a DELL Inspiron 15-5557, which has 16 GB of RAM, Intel Core i7 2.5 GHz processor and Ubuntu 16.04 LTS operating system.
- LSDi Server computer used to run a server broker in experiments using the Internet. It has 32 GB of RAM and an Intel Xeon processor 1.7 GHz. This broker can be accessed using the following url: www.lsdi.ufma.br:1883.

Regarding the reliability of delivery configurations, the experiments vary in three types:

- Experiments with reliability set to level 0: in this experiment the **at most once**.
- Experiments with reliability set to level 1: In this experiment the **at least once** policy was employed.
- Experiments with reliability set to level 2: In this experiment the policy **exacly once** was used.

In this experiment, it is important to note that only Delivery Reliability and Session policies were enabled. The other QoS policies were disabled and, therefore, did not affect the experiment performance.

In relation to the types of network used, the experiments varied in three types:

- Experiments without network usage: In this experiment no network connection was used. Publisher and subscriber were connected to a micro broker running on the same device as the test application (the smartphone).
- Experiments using local network: in this experiment a Wireless Local Area Network (WLAN) network was used. Publisher and subscriber were connected to an external server broker. In this case, the server broker ran on the notebook, which also acted as a network access point. The WLAN was formed only by the smartphone and the notebook, and there was no connection to the Internet.
- Experiments using fixed Internet: in this experiment, a local Wifi network was used. Internet access was made via a wireless Asymmetric Digital Subscriber Line (ADSL) router/modem. Both publisher and subscriber were connected to a server broker running on the LSDi server.
- Experiments using mobile Internet: in this experiment was used a 4G connection. Both publisher and subscriber were connected to a server broker running on the LSDi server.

It is important to note that in the experiments using the Internet, the test application ran on a network distinct from the one in which the broker was running. The LSDi server was connected to the institutional network of the Federal University of Maranhão, located in the city of São Luís, while the test application was connected to the Internet using the services of a telephone/band operator called Oi (http://www.oi.com.br/), in the city of São José de Ribamar. Both cities are part of a metropolitan region called São Luís Island, in the State of Maranhão. The distance between these cities is approximately 20 km.

For each type of network, all possible reliability levels were tested. Therefore, experiments were carried out with 12 different configurations. Considering that each type of experiment/configuration was repeated 5 times, a total of 60 executions were performed. Table 2, Table 3 and Table 4 show respectively the results obtained in relation to the communication time, processing time and total delivery time, taking into account only the average obtained with the repetitions of each setting. The average, maximum, minimum and standard deviation for each of the defined metrics were also calculated. We also define that the representative performance of each type of network is given by the average obtained considering the three levels of reliability tested.

In relation to experiments without network use, it can be observed that the average communication time using the level 0 reliability (62.25 ms), practically doubles by raising the level of reliability to 1 (reaching 186.17 ms) and triple when fraising the reliability level to 2 (reaching 236.68 ms). Meanwhile, the average data delivery time using level 0 reliability (99.46 ms), practically doubles by raising the reliability level to 1 (reaching 217.51 ms) and triples by raising the reliability level to 2 (reaching 271.64 ms). As expected, level 0 reliability performed better than levels 1 and 2 because it did not require confirmation of message delivery. Level 1 has performed better than level 2 because the delivery confirmation mechanism used by the first (simple handshake) requires fewer message exchanges than the second (double handshake). Thus, the performance of the micro broker degrades as the level of reliability used increases, because the higher the level of reliability, the more memory and processing resources are required.

In relation to the experiments with the use of isolated local network, which obtained the best average performance, it can be observed that, as in the experiments without the use of the network, and also for the same reasons, the communication time and the total data delivery time using level 0 reliability (74.37 ms and 112.46 ms, respectively) were also lower than those using levels 1 (in which the communication time was 92.51 ms and the total data delivery time was 128.60 ms) and 2 (in which the communication time was 126.23 ms and the total data delivery time was 162.69 ms). However, the difference between the communication times, and consequently between the total data delivery times, using the levels 0 and 2, is much lower than those obtained in the experiments without the use of the network. This time, as far as communication time is concerned, the difference between levels 0 and 1, as well as the difference between levels 1 and 2, is less than 25%. Regarding total delivery time, the difference between levels 0 and 1 is less than 25%, while the difference between levels 1 and 2 is less than 30%. The reason why this occurs is strongly related to the type of broker that each one uses. While in the experiments without using the network a micro broker was used (executing in the mobile device), in the experiments using the local network a server broker executing in the notebook was used. The difference in processing power and memory available in both execution platforms causes the micro broker’s performance to deteriorate faster than the server broker when reliability increases, causing the latter to send messages to the recipients faster than the first. This difference in performance in favor of the server broker is almost always enough to compensate for the delay imposed by the isolated local network communication. The exception occurs when both types of experiments use reliability level 0. In this case, thanks to the publication fee used, the cost of processing the messages (which do not require delivery confirmation) is so low that the delay imposed by the isolated local network has become more significant than the cost of processing carried out by the broker, resulting that the time of communication and the total time of delivery of the data obtained in the experiments without use of the network, for this level of reliability, are the smallest among all the types of experiments performed.

In both cases, the communication times obtained using level 0 reliability (233.35 ms using Wifi and 262.55 ms using 4G) practically double when using level 1 reliability (reaching 417.04 using Wifi and 472.12 using 4G) and triples by raising reliability to level 2 (681.42 using Wifi and 807.14 using 4G). The total data delivery times, using level 0 reliability (267.14 ms using Wifi and 298.97), increased about 60% by raising the reliability level to 1 (reaching 440.07 ms using Wifi and 506.72 using 4G). The times obtained using level 2 reliability increased about 60% from level 1 (reaching 715.80 ms using Wifi and 844.15 using 4G). As expected, the delay imposed by the Internet meant that these experiments had inferior performances in relation to other network configurations.

Analyzing all types of experiments, it can be observed that the performance of the middleware in the scenarios tested in relation to the average data processing time before sending and after receiving the data is always low, close to 35 ms, with small variations. In general, we can conclude that the processing time imposed by the middleware has a considerably smaller impact on the total data delivery time than the communication time (transmission delay + processing time in the broker). The observed communication times for all network configurations, even in experiments using the Internet, whose results tend to vary according to the state of the network, allow us to conclude that, under similar network conditions, the M-Hub/CDDL offers communication with low *overhead*, allowing the transmission of sensor data in real time, thanks to the adoption of a protocol optimized for IoT.

### 5.2. Evaluating of the Message Delivery Reliability

Mobile applications for IoT are generally subject to context data loss during the delivery process due to intermittent connectivity and other types of failures. The objective of this experiment is to verify the efficiency of the reliability policy implemented by M-Hub/CDDL in scenarios of intermittent connectivity, which varied in two basic types:

- Low-intermittency scenario: in which the connectivity to the broker was programmatically interrupted within a range that varied randomly according to a uniform distribution in the interval between 120 and 180 s.
- High-intermittence scenario: in which connectivity with the broker was programmatically interrupted within a range that varied randomly according to a uniform distribution in the interval between 12 and 18 s, so the intermittence rate was 10 times greater than the one in the previous scenario.

In both scenarios, the duration of the disconnection varied randomly between 3 and 6 s, according to a uniform distribution. We used the same test application from the previous scenario, as well as the same hardware. Again, each experiment lasted approximately 30 min and the message delivery rate was 1 Hz. Failures were generated by shutting down the wireless network interface, causing the TCP/IP connection between the test application and the broker to close unexpectedly. The scenarios considering low and high intermittency were tested using two of the network configurations employed in the previous experiments: isolated local area network and Internet through Wifi. Variations were also promoted in relation to the three levels of reliability offered by the middleware. As in the previous experiment, only Delivery Reliability and Session policies were used. The other QoS policies were disabled and, therefore, did not affect the experiment performance. Each experiment was repeated 5 times, totaling 20 experiments. The defined evaluation metric was the message loss percentage obtained in each scenario. The results are presented in Table 5.

It is possible to observe that the middleware was able to deliver 100% of messages in all situations where the reliability level employed was greater than zero, allowing to conclude that the M-Hub/CDDL has a high degree of delivery efficiency when using such reliability configurations. This performance was obtained due to the combination of the default delivery confirmation mechanism provided by MQTT, coupled with the intermediate buffer mechanism implemented by M-Hub/CDDL. The latter increases reliability for two reasons: (i) unlike the buffer provided by the default configuration of MQTT clients, the intermediate buffer stores all published messages that require delivery confirmation. This allows the middleware to resend to the broker all messages whose deliveries have not been committed after a given period; (ii) contrary to what MQTT clients usually do by default (they stop trying to resend lost messages as soon as the connection expires), the M-Hub/CDDL continues trying to resend messages stored in the intermediate buffer as soon as a new connection is established.

It is also possible to observe that the average message loss rate in the scenario of high intermittency using level 0 reliability on the Internet (35.5%) is significantly higher than that presented by the local network in the experiment that used the same intermittency rate and the same reliability level (23.8%). The difference is practically 12%. This significant difference is attributed to the following facts: (i) local network communication is typically more reliable and less subject to failure than in long-distance networks; (ii) low intermittency forced the application to reconnect more often to the broker. Considering that the observed local connection time (which ranged from 9 to 10 ms) was about 30 to 50 times greater than the Internet connection time (which ranged from 300 to 500 ms), the test application spent more time connected to the broker in the experiments using local network than in those using the Internet, allowing that the first delivered a greater number of messages than the second. However, the connection time does not have a big impact if the application does not have to reconnect to the broker so many times. For this reason, the loss rates for both types of network configuration are very close in the low intermittent scenarios.

Finally, the low standard deviation presented allows us to conclude that the results tend to remain stable for IoT applications that perform under conditions similar to those simulated in this experiment.

### 5.3. Evaluation of Monitoring Time

The monitoring time, i.e., the time taken to process the information in order to determine if there was any kind of context or QoC variations, needs to be low. Otherwise, if significant oscillations occur, they will be perceived very late by the consumer application. Consequently, this may prevent the application from reacting to such changes in a timely manner.

In order to evaluate the performance of the M-Hub/CDDL in relation to the monitoring time, a single type of experiment was conducted in which the arrival rate of messages (generated synthetically by a virtual sensor) was 1 message per millisecond, a frequency that can be considered relatively high. In each experiment, 1800 messages were published. Of this total, 180 samples had accuracy values greater than zero, while the others had values equal to zero. The monitoring rule used in this experiment consisted of detecting the arrival of samples whose values were greater than zero (“Select * from SensorDataMessage where accuracy> 0.0”). Every 10 ms, a sample with different accuracy was sent, simulating a QoC variation. Each experiment was repeated 5 times, always using the local micro broker (without network use) and level 0 reliability. Except for reliability, all other policies were disabled and, therefore, did not affect the experiment performance. The smartphone that ran the test application was the same as the previous experiments. Monitoring time considered is the interval between the arrival of the message at the subscriber and the notification of the QoC variation to the consumer application. Thus, neither the network configuration nor the level of reliability chosen interfered with the results obtained.

The average monitoring time was 12.49 ms. The maximum monitoring time reached was 14.53 ms, while the minimum time was 10.15 ms, generating a standard deviation of 1.56 ms. These results allow us to conclude that M-Hub/CDDL monitoring time is quite low, and the standard deviation obtained in the experiment indicates that this performance tends to remain stable if it is repeated in similar conditions. It is important to note that the execution of the CEP rules did not compromise the performance of the test application, that is, the middleware remained stable throughout the period of the experiments, even with a high event arrival rate.

### 5.4. Evaluation of Memory Consumption

The objective of this experiment was to measure the amount of memory allocated to the middleware components responsible for QoC monitoring and QoC evaluation, both based on the use of complex event processing (CEP) agents, as explained in Section 3.7. The same test application used in the previous experiments was used, only changing the publication rate, which in this experiment was 1 message per second (a frequency considered moderate). For this experiment, all QoS policies were disabled and, therefore, did not affect the experiment performance. The application was run for 30 min. From the use of the Android Monitor tool (built into the Android Studio) data regarding memory consumption were collected in 5 instants of application execution: 5, 10, 15, 20 and 25 min. Table 6 shows the average results obtained considering 5 repetitions of the experiment.

From these results, it is possible to observe that the QoC monitoring and QoC evaluation components require a little amount of memory to process data flows with a moderate frequency and that this amount remains stable during the entire application execution period.

### 5.5. Evaluation of Battery Consumption

The objective of this experiment was to evaluate the battery consumption by the middleware components. For this, we used the same test application of the previous experiment and the same data publication rate. The application was run 5 times for 12 h each, and the battery level was measured at the beginning and at the end of the execution. It is important to note that while the application was running the device screen remained turned off. In addition, with the exception of operating system processes, there were no other processes running on the device. As in the previous experiment, all QoS policies were disabled and, therefore, did not affect the experiment performance. As shown in Table 7, the average battery consumption was 15.6% . This average consumption is considered to be low, indicating that middleware can be used to publish data and monitor QoC on conventional mobile devices.

## 6. Comparative Analysis and Discussions

Analyzing the case study and experiments performed, as well as the functionalities implemented by the various components of the proposed middleware, it is concluded that M-Hub/CDDL fulfills all the requirements defined in Section 3.3 and therefore fulfills the purpose for which it was built, which is to facilitate the development of IoT/IoMT applications, especially those that have multiple management and quality of context requirements.

This section addresses two discussion points. Initially, the proposed middleware is positioned in relation to the reference architectures for IoT analyzed by Calvalcante et al. [13]. Subsequently, a comparative analysis is performed between the M-Hub/CDDL and the middleware systems with QoC support presented in Section 2, taking into account the open issues that have been raised.

In relation to the comparison with reference architectures for IoT, it is noted that M-Hub/CDDL is closer to WSO2 (version 0.9.0) [19] than other architectures. Both have several common requirements, such as context awareness, support for device heterogeneity, sensor discovery and management, high-level programming interfaces, event processing, among others. Another similarity between the proposed middleware and the last version of WS02 is that both adopt MQTT as the standard communication protocol between the devices and the cloud. WSO2 recommends the use MQTT over WebSockets because it architecture follows the Web of Things model. M-Hub/CDDL, on the other hand, depending on the platform on which the CDDL client executes, can use both the standard MQTT (on mobile and desktop CDDL clients) and MQTT over WebSockets (on CDDL web client). As explained in Section 3, the standard MQTT version is used by desktop and mobile CDDL clients, whereas the MQTT over WebSockets version is used by CDDL web clients. Therefore, in addition to meeting the requirements of the traditional IoT model, the proposed middleware also encompasses WoT requirements. It is important to note that WSO2 recommends HTTP as an alternative communication protocol to MQTT, while M-Hub/CDDL does not have a second device-to-cloud communication protocol option. Another difference is that WSO2 does not define how producers and consumers running on the same mobile device can communicate without using the network. M-Hub/CDDL solves this problem using MQTT and a Micro Broker that mediates communication between producers and local consumers. However, the main difference between M-Hub/CDDL and WSO2 is that this reference architecture, unlike the proposed middleware, does not address aspects of QoC provisioning, evaluation, and monitoring.

With respect AWARENESS, COSMOS, COPAL, INCOME and SALES middleware systems, Table 8 summarizes a comparison between them and the M-Hub/CDDL emphasizing the described open research issues.

Regarding quality of context provisioning, unlike the middleware systems presented, M-Hub/CDDL is not limited to the provisioning of a specific class of QoC parameters. Instead, the QoC support offered by the proposed middleware comprises both QoI parameters and QoS policies. Thus, M-Hub/CDDL is a more suitable middleware system for applications that have multiple QoC requirements.

Concerning QoC monitoring, M-Hub/CDDDL advances over the other middleware systems presented because it provides mechanisms that allow the context-aware application to specify QoC variations events from which it requires to be notified in near real time. Therefore, compared to middleware systems, M-Hub/CDDL may be considered more suitable for applications that need to dynamically adapt to changes in quality of context.

In relation to the support for interaction with heterogeneous physical sensors, most of the middleware described only provide a programming interface to mask the data source using sensors adapters/wrappers. In this case, it is up to the programmer to implement the device-specific adapters/wrappers and the lower-level mechanisms responsible for the discovery of nearby sensors and direct acquisition of raw data, which would require considerable effort. M-Hub/CDDL and COPAL stand out in this respect since both offer not only abstractions for the sensor hardware heterogeneity, but also provide mechanisms that facilitate these tasks, even though they are focused on devices with different characteristics. In the case of COPAL, the service descriptions is based on the UPnP standard. However, papers presenting the COPAL middleware do not discuss issues related to how to describe device services taking into account the quality of the information provided by the sensors. In the case of M-Hub/CDDL, sensor management is a requirement implemented by the S2PA Service. This service already provides native support for interaction with sensors using Bluetooth Classic and BLE technologies. However, programmers can extend the middleware to implement support for other communication technologies. M-Hub/CDDL’s services description takes into account the quality of the information provided by the sensors, as well as the quality of service offered by the context service provider. Thus, QoC (both QoI and QoS) becomes a criteria that can be used in service discovery queries. In addition, M-Hub is able to download sensors adapters/wrappers from a Web repository and load them dynamically. This ability is relevant in IoMT scenarios because it allows the mobile gateway to get the sensor adapters/wrappers opportunistically.

Another advancement in middleware systems presented, including those that have some kind of QoS support, is that M-Hub/CDDL addresses the challenge of providing reliability in mobile environments. It is important to highlight that the proposed middleware, in comparison to the other solutions described, is the only one that adopts an optimized IoT protocol: MQTT. Thanks to the use of this protocol combined with the intermediate buffering mechanism of the CDDL, the proposed middleware becomes the most appropriate solution for IoT applications that, in addition to other QoS parameters, also require delivery reliability in unstable networks.

Considering the way in which each presented solution was evaluated, it is noticed that M-Hub/CDDL and SALES are the only middleware systems with QoC support whose evaluation was not restricted to case studies alone. Both had performance/efficiency of the distribution service quantitatively assessed. However, the M-Hub/CDDL evaluation is more comprehensive, as it also addresses measurement aspects of resource consumption in mobile devices.

Given the above, it can be concluded that, in general, M-Hub/CDDL presents significant advances in relation to other middleware solutions with QoC support, since it addresses the challenges presented more consistently.

## 7. Conclusions and Future Work

Quality of Context (QoC) is a requirement imposed by emerging services and applications for the Internet of Things (IoT) that need to be aware of the user context and their environment, as well as of the quality of the data provided by sensors and/or the quality of the distribution service of this data through different types of networks. In IoMT scenarios, the unrestricted mobility of smart objects with limited resources leads to the use of mobile gateways to enable discovery, opportunistic interaction, and data acquisition via WPAN, as well as an efficient data distribution layer capable of delivering the context information to consumers with the quality level they require, since QoC has a direct impact on the user experience of context aware applications.

One of the most important quality of service requirements is the delivery reliability, that is, the ability to ensure that the data reaches its destination. The refresh rate parameter allows applications to send and receive data according to their processing and memory capabilities, which on mobile devices are more scarce resources. Regarding quality of information, which can be directly affected by QoS, parameters such as age, validity time, resolution, and accuracy can help determine the relevance that context information will have for a particular application. Considering that QoC may oscillate over time, it is important that context-aware applications have at their disposal a monitoring mechanism capable of detecting changes in quality of context in a timely manner. In this way, these applications can quickly perform actions in response to these variations.

One of the contributions of this work was a state-of-the-art survey regarding QoC-supported context middleware systems for IoT applications. This survey corresponded to an important contribution of this article because, to the best of our knowledge, no literature review had focused on analyzing how these tools meet application QoC requirements. Based on this survey, a comparative analysis was carried out between the known middleware systems and the M-Hub/CDDL, taking into account different aspects of comparison. It was possible to conclude that the support offered by the proposed middleware is more comprehensive and complete than that provided by other tools analyzed. Regarding QoC, in addition to providing more context quality parameters and policies, M-Hub/CDDL is able to meet both QoI and QoS requirements, unlike other solutions that are focused on provisioning one or other quality dimensions, something that is reflected in the monitoring support, since the mechanisms implemented for detecting QoC variations are focused on the provided parameters. In addition, most of the work related to other context middleware systems do not address other relevent aspects highlighted in the comparative analysis, such as the support for interaction with physical sensors and the reliable delivery of context data in mobile environment.

As another contribution of this work, a new context middleware, called M-Hub/CDDL, was presented aimed at the development of IoT/IoMT applications, especially those that require quality of context. This middleware, which combines the use of a mobile gateway with a data distribution service, was designed to meet a wide range of requirements, such as: (i) interaction with physical sensors; (ii) local and remote data distribution using multiple connections; (iii) registration and discovery of services through queries; (iv) unified programming model; (v) provisioning and monitoring of various quality of context (QoI and QoS) parameters.

The performance evaluation of the M-Hub/CDDL’s data distribution service led us to the conclusion that the data processing time of the middleware components is relatively low, and a small impact on the total delivery time required for communication between data producers and consumers. This communication time varies mainly according to the type of network configuration, the type of broker and the level of reliability employed. For all the network configurations in which the middleware was evaluated, the performance obtained by it was considered satisfactory. In relation to the evaluation of the reliability of the data delivery to the consumer, the combination of the message loss detection mechanism of the MQTT protocol, which provides three levels of reliability, combined with the intermediate buffer mechanism of the M-Hub/CDDL, which stores and returns lost messages as soon as the connection is reestablished, has achieved 100% of successful messages delivery in different evaluation scenarios that have simulated high and low intermittent connection with the broker. The performance of the monitoring mechanism was also satisfactory, since the time it takes to process events, even with a high data arrival rate, is a few milliseconds. The efficiency of this mechanism is related to the integration of the middleware with a mobile-based CEP Engine, responsible for the execution of complex event processing. On the other hand, the middleware most computing intensive components (QoS Evaluator and Monitor) present low memory and battery consumption. These results reinforce the thesis that the middleware is suitable for use on commonly used mobile devices, allowing the context aware application to publish and monitor data without significantly compromising the device’s battery life.

Finally, as last contribution, a case study was presented allowing us to verify the middleware suitability to the development of a mobile application focused on patients monitoring. Since the middleware has satisfactorily met the requirements imposed by the target application, it is possible to generalize that the M-Hub/CDDL can be used for a series of IoT applications with similar characteristics.

Future work involves the ability to perform semantic processing of context data considering large data flows. Another requirement to be explored is a built-in support for high level queries useful in IoT, such as the ones considering the smart objects movement patterns.

## Figures and Tables

**Table 1:** Comparison between related work emphasizing the open research issues.

Middleware	Quality of Context Provisioning	Quality of Context Monitoring	Heterogeneous Sensors Management	Reliable Data Delivery in Mobility Scenarios
AWARENESS	Focus on QoI, but with some support for QoS.	Not addressed.	Provides only an API for implementing adapters/wrappers for the sensors drivers.	Not addressed
COSMOS	Focus on QoI, only.	Not addressed.	Provides only API for implementing adapters/wrappers for the sensors drivers.	Not addressed.
COPAL	Greater focus on QoI, but with some QoS support.	Not addressed.	Provides an API for implementing specific adapters/wrappers for the sensors drivers; Provides a sensors management service. Service description is based on the UPnP.	Not addressed.
INCOME	Focus on QoI, only.	Not addressed.	Provides only API for implementing adapters/wrappers for the sensors drivers.	Not addressed.
SALES	Focus on QoS, only.	Not addressed.	Provides only API for implementing adapters/wrappers the sensors drivers.	Not addressed.

**Table 2:** Evaluation of the Distribution Service Performance in relation to the Communication Time.

Configuration	Reliability	Average (ms)	Maximum (ms)	Minimum (ms)	Standard Deviation (ms)
Without Network (Micro Broker)	0	62.25	66.61	59.60	3.20
Without Network (Micro Broker)	1	186.17	194.99	177.92	6.45
Without Network (Micro Broker)	2	236.68	241.77	232.87	3.35
**Average of Experiments without Network Usage**		**161.70**	**167.79**	**156.80**	**4.33**
Isolated Local Network	0	74.37	80.79	71.22	3.94
Isolated Local Network	1	92.51	99.92	88.59	4.60
Isolated Local Network	2	126.23	131.18	121.82	4.48
**Average of Experiments using Isolated Local Network**		**97.70**	**103.97**	**93.88**	**4.34**
Fixed Internet (Wifi + ADSL)	0	233.35	241.40	228.61	5.56
Fixed Internet (Wifi + ADSL)	1	417.04	490.48	375.22	46.57
Fixed Internet (Wifi + ADSL)	2	681.42	711.17	666.79	17.64
**Average of Experiments using Fixed Internet**		**443.94**	**481.01**	**423.54**	**23.26**
Mobile Internet (4G)	0	262.55	273.69	254.81	7.98
Mobile Internet (4G)	1	472.12	483.05	456.64	9.98
Mobile Internet (4G)	2	807.14	845.54	782.28	25.36
**Average of Experiments using the 4G Internet**		**513.94**	**534.09**	**497.91**	**14.44**

**Table 3:** Evaluation of the Distribution Service Performance in relation to the Processing Time.

Configuration	Reliability	Average (ms)	Maximum(ms)	Minimum(ms)	Standard Deviation (ms)
Without Network (Micro Broker)	0	37.21	42.05	34.01	4.21
Without Network (Micro Broker)	1	31.34	36.91	26.15	3.88
Without Network (Micro Broker)	2	34.95	37.66	32.84	2.26
**Average of Experiments without Network Usage**		**34.50**	**38.87**	**31.00**	**3.45**
Isolated Local Network	0	34.66	37.20	32.28	2.13
Isolated Local Network	1	37.14	39.44	34.36	1.83
Isolated Local Network	2	36.46	42.37	34.24	3.39
**Average of Experiments using Isolated Local Network**		**36.09**	**39.67**	**33.63**	**2.45**
Fixed Internet (Wifi + ADSL)	0	33.79	37.34	31.75	2.31
Fixed Internet (Wifi + ADSL)	1	34.60	36.20	33.68	0.97
Fixed Internet (Wifi + ADSL)	2	35.97	40.06	33.28	3.11
**Average of Experiments using Fixed Internet**		**34.79**	**37.87**	**32.90**	**2.13**
Mobile Internet (4G)	0	36.42	42.83	34.27	3.64
Mobile Internet (4G)	1	34.60	36.20	33.68	0.97
Mobile Internet (4G)	2	34.37	36.92	32.14	2.35
**Average of Experiments using the 4G Internet**		**35.13**	**38.65**	**33.36**	**2.32**

**Table 4:** Evaluation of the Distribution Service Performance in relation to the Total Time of Information Delivery to the Consumer.

Configuration	Reliability	Average (ms)	Maximum (ms)	Minimum (ms)	Standard Deviation (ms)
Without Network (Micro Broker)	0	99.46	108.19	93.60	7.35
Without Network (Micro Broker)	1	217.51	225.84	204.07	8.89
Without Network (Micro Broker)	2	271.64	278.91	266.51	5.31
**Average of Experiments without Network Usage**		**196.20**	**204.31**	**188.06**	**7.18**
Isolated Local Network	0	112.46	120.45	105.30	6.52
Isolated Local Network	1	128.60	134.85	123.47	4.24
Isolated Local Network	2	162.69	167.02	156.37	4.49
**Average of Experiments using Isolated Local Network**		**134.58**	**140.77**	**128.38**	**5.08**
Fixed Internet (Wifi + ADSL)	0	267.14	278.74	260.41	7.73
Fixed Internet (Wifi + ADSL)	1	440.07	472.06	412.94	24.68
Fixed Internet (Wifi + ADSL)	2	715.80	743.30	703.24	16.59
**Average of Experiments using Fixed Internet**		**474.33**	**498.03**	**458.86**	**16.33**
Mobile Internet (4G)	0	298.97	309.12	289.59	9.24
Mobile Internet (4G)	1	506.72	519.25	490.32	10.77
Mobile Internet (4G)	2	844.15	882.05	822.06	23.86
**Average of Experiments using the 4G Internet**		**549.95**	**570.14**	**533.99**	**14.62**

**Table 5:** Results from the Evaluation of Message Delivery Efficiency in Intermittent Connection Scenarios.

Network Configuration	Reliability	Interval between Disconnections (s)	Average Loss Rate	Maximum Loss Rate	Minimum Loss Rate	Standard Deviation Loss Rate
Isolated Local Network	0	120–180	3.8%	4.2%	3.6%	0.3%
Isolated Local Network	0	12–18	23.8%	28.6%	21.1%	2.9%
Isolated Local Network	1	120–180	0.0%	0.0%	0.0%	0.0%
Isolated Local Network	1	12–18	0.0%	0.0%	0.0%	0.0%
Isolated Local Network	2	120–180	0.0%	0.0%	0.0%	0.0%
Isolated Local Network	2	12–18	0.0%	0.0%	0.0%	0.0%
Internet (Wifi + ADSL)	0	120–180	3.8%	3.9%	3.5%	0.2%
Internet (Wifi + ADSL)	0	12–18	35.5%	36.3%	33.9%	1.0%
Internet (Wifi + ADSL)	1	120–180	0.0%	0.0%	0.0%	0.0%
Internet (Wifi + ADSL)	1	12–18	0.0%	0.0%	0.0%	0.0%
Internet (Wifi + ADSL)	2	120–180	0.0%	0.0%	0.0%	0.0%
Internet (Wifi + ADSL)	2	12–18	0.0%	0.0%	0.0%	0.0%

**Table 6:** Memory consumption in Kilobyte (Kbytes).

	5 min	10 min	15 min	20 min	25 min	Average	Standard Deviation
**QoC Evaluator**	263	262	268	269	270	266	2.77
**Monitor**	213	213	216	219	280	214	3.14

**Table 7:** Battery Consumption.

	Minimum	Maximum	Average	Standard Deviation
**Battery Consumption**	15%	16%	15.6%	0.55

**Table 8:** Comparison between related work and M-Hub/CDDL emphasizing the open research issues.

Middleware	Quality of Context Provisioning	Quality of Context Monitoring	Heterogeneous Sensors Management	Reliable Data Delivery in Mobility Scenarios
M-Hub/CDDL	Provides multiple QoI parameters and QoS policies.	Monitors various QoI parameters and QoS policies.	Provides an API for implementing specific adapters/wrappers for sensors drivers;	Provides support for multiple reliability levels;
			Provides a generic and extensible sensors management service with native support for Bluetooth Classic and BLE technologies;	Provides an intermediate buffer which increases the reliability delivery;
			Provides support for the sensor dynamic loading from the web software repositories.	Provides support also for disconnected operations and IP address exchange.
AWARENESS	Focus on QoI, but with some support for QoS.	Not addressed.	Provides only an API for implementing adapters/wrappers for the sensors drivers.	Not addressed.
COSMOS	Focus on QoI, only.	Not addressed.	Provides only API for implementing adapters/wrappers for the sensors drivers.	Not addressed.
COPAL	Greater focus on QoI, but with some QoS support.	Not addressed.	Provides an API for implementing specific adapters/wrappers for the sensors drivers;	Not addressed.
			Provides a sensors management service. Service description is based on the UPnP.	
INCOME	Focus on QoI, only.	Not addressed.	Provides only API for implementing adapters/wrappers for the sensors drivers.	Not addressed.
SALES	Focus on QoS, only.	Not addressed.	Provides only API for implementing adapters/wrappers the sensors drivers.	Not addressed.
